# High Genomic Variability in Equine Infectious Anemia Virus Obtained from Naturally Infected Horses in Pantanal, Brazil: An Endemic Region Case

**DOI:** 10.3390/v12020207

**Published:** 2020-02-12

**Authors:** Camila Dantas Malossi, Eduardo Gorzoni Fioratti, Jedson Ferreira Cardoso, Angelo Jose Magro, Erna Geessien Kroon, Daniel Moura de Aguiar, Alice Mamede Costa Marque Borges, Marcia Furlan Nogueira, Leila Sabrina Ullmann, João Pessoa Araujo

**Affiliations:** 1 Institute for Biotechnology, São Paulo State University (Unesp), Botucatu 18607-440, Brazil; camilamalossi@gmail.com (C.D.M.); angelo.magro@unesp.br (A.J.M.); ullmannleila@gmail.com (L.S.U.); 2 Agrarian Sciences Institute (ICA), Vales do Jequitinhonha e Mucuri Federal University (UFVJM), Unaí 39803-371, Brazil; efioratti@yahoo.com.br; 3 Center for Technological Innovation, Evandro Chagas Institute, Ananindeua 67030-000, Brazil; jedson.cardoso@gmail.com; 4 Virology Laboratory, Universidade Federal de Minas Gerais, Belo Horizonte 31270-901, Brazil; ernagkroon@gmail.com; 5 Virology and Rickettsiosis Laboratory, Mato Grosso Federal University, Cuiabá 78060-900, Brazil; danmoura@ufmt.br (D.M.d.A.); alicemmarques@hotmail.com (A.M.C.M.B.); 6 EMBRAPA Pantanal, Corumbá 79320-900, Brazil; marcia.furlan@embrapa.br

**Keywords:** equine infectious anemia virus, molecular characterization, endemic region, RNA genome, equine

## Abstract

Equine infectious anemia virus (EIAV) is a persistent lentivirus that causes equine infectious anemia (EIA). In Brazil, EIAV is endemic in the Pantanal region, and euthanasia is not mandatory in this area. All of the complete genomic sequences from field viruses are from North America, Asia, and Europe, and only proviral genomic sequences are available. Sequences from Brazilian EIAV are currently available only for *gag* and LTR regions. Thus, the present study aimed for the first time to sequence the entire EIAV genomic RNA in naturally infected horses from an endemic area in Brazil. RNA in plasma from naturally infected horses was used for next-generation sequencing (NGS), and gaps were filled using Sanger sequencing methodology. Complete viral genomes of EIAV from two horses were obtained and annotated (Access Number: MN560970 and MN560971). Putative genes were analyzed and compared with previously described genes, showing conservation in *gag* and *pol* genes and high variations in LTR and *env* sequences. Amino acid changes were identified in the p26 protein, one of the most common targets used for diagnosis, and p26 molecular modelling showed surface amino acid alterations in some epitopes. Brazilian genome sequences presented 88.6% nucleotide identity with one another and 75.8 to 77.3% with main field strains, such as EIAV Liaoning, Wyoming, Ireland, and Italy isolates. Furthermore, phylogenetic analysis suggested that this Brazilian strain comprises a separate monophyletic group. These results may help to better characterize EIAV and to overcome the challenges of diagnosing and controlling EIA in endemic regions.

## 1. Introduction

Equine infectious anemia virus (EIAV) is a lentivirus with an almost worldwide distribution that infects equids, such as horses, mules, and donkeys. EIAV causes a persistent infection characterized by recurring febrile episodes associated with viremia, fever, thrombocytopaenia, and wasting clinical signs. Most animals progress from a chronic stage characterized by recurring peaks of viremia and fever to an asymptomatic stage of infection, and asymptomatic carriers remain infective for life. Equine infectious anemia (EIA) is transmitted by blood-sucking insects, mainly horseflies and stable flies, as well as by contaminated syringes and needles, blood-contaminated instruments, and contaminated blood used in transfusions. No effective treatment or vaccine is currently available [1,2].

EIAV has a single-stranded RNA genome of approximately 8.2 kb and contains three major genes, *gag* (structural), *pol* (enzymes), and *env* (surface glycoproteins), which are flanked by long terminal repeats (LTRs). EIAV is a complex lentivirus and encodes three accessory genes: *tat*, *rev*, and *S2* [1,2]. One of the main resistance mechanisms of lentiviruses is their ability to evolve based on the response of the host’s immune system. Moreover, it is an evolutionary characteristic of these viruses that the mutation rate correlates directly with the viral replication levels and with the virus polymerase error rate. EIAV reverse transcriptase (RT) is as prone to errors as any other RT, and incorrect purine-pyrimidine pairing occurs more often than does incorrect purine-purine pairing [3]. The *gag* and *pol* genes are generally the most conserved of the genome, whereas *env* and *rev* are the most variable [1]. The core protein p26, encoded by the *gag* gene, presents a high conservation rate, and this antigen is the basis for the majority of serological tests commercially available for the detection of EIAV-infected equids [2].

Studies suggest that cycling episodes observed during the EIA chronic phase are associated with distinct viral populations that can be characterized as genomic and/or antigenic variants. These observations suggest that the nature of the chronic phase is linked to the production and selection of the viral envelope (*env*) or immunological escape mutants [4]. Antigenic escape mutants are viruses that cause persistent infections due to mutations in surface proteins, allowing replication even in hosts that produce antibodies against EIAV [5]. These conclusions are supported by analysis of the viral surface protein (SU-gp90), which shows amino acid substitutions in eight hypervariable domains in infected horses and ponies that exhibit cyclic clinical episodes [6]. These substitutions in SUs of different strains are distributed throughout the protein, except in the amino-terminal region. Another conserved characteristic is the presence of cysteines, suggesting that disulfide bonds are essential for the structural and functional integrity of the SU [2].

Genomic viral sequencing has been applied to study viral variants, quasispecies, and protein changes in lentiviruses. Characterization of viral populations in early HIV infection, analysis of viral evolution patterns, and identification of super-infective strains have been studied by next-generation sequencing (NGS), also known as high-throughput sequencing (HTS) [7]. Complete genomic sequences of field-isolated EIAV from five different countries have been published to date: EIAV_WY_ (United States of America), EIAV_LIA_ (China), EIAV_MIY_ (Japan) [8], EIAV_IRE_ (Ireland) [9], EIAV_DEV_ and EIAV_CORN_ (England) [10], and the incomplete sequence EIAV_ITA_ (Italy) [11]. Only the last and most recently obtained sequences, EIAV_ITA,_ EIAV_DEV_ and EIAV_CORN_, were obtained by NGS. The other four complete genomic sequences were obtained by Sanger sequencing. Quasispecies identification and intrahost variations cannot be analyzed by the Sanger method, thus, early studies are limited. The sequences from Italy and Ireland are very similar to each other. Sequences from China, Japan, the United States of America, England, and Italy/Ireland share approximately 80% identity, forming five separated clades and suggesting an independent evolution from a common ancestor [2,10,11,12]. This fact indicates that the establishment of similar approaches for molecular diagnosis methods and control programs in different regions might be difficult [2].

As in other countries, EIA diagnosis in Brazil is based on serological exams for viral antibody detection. The diagnosis is primarily based on agar gel immunodiffusion (AGID) with an enzyme-linked immunosorbent assay (ELISA) as a secondary test [13]. In the Brazilian Pantanal region, considered a high-risk area for EIA transmission, euthanasia of EIA-positive animals is not mandatory because equids do not leave the owners’ facilities. Studies in southern Pantanal from 1990 to 1995 [14] revealed a mean prevalence of 18.17% in working equids, which are the kind of horses that are regularly handled. Recently, it was reported that seropositivity among equids used for cattle management increased to 38.6% [15]. In the northern Pantanal municipality of Poconé, MT, it was verified that 31.5% of working equids are infected [16]. Therefore, the situation in the Pantanal region is of particular concern, due to the important role of equids in local livestock production, in addition to the economic losses associated with the disease [17].

A circulating EIAV in an endemic region with different control strategies, such as Pantanal, can potentially present unique characteristics considering the high mutation rate in lentiviruses, and equids remain infected with the virus for years and act as a source of infection to other animals. Virus introduction occurred in Pantanal in 1974 [18], and EIAV has circulated in this region for almost 50 years, a length of time sufficient for differentiation in relation to other viruses found in other countries [19]. In addition, the possible association between a cyclic clinical condition and the generation of distinct viral populations in infected equines indicate that positive animals remaining in the population might provide increased EIAV genetic diversity in the Pantanal region [20].

In this study, we aimed to characterize circulating EIAV in the Pantanal region by sequencing the complete viral genome. Our results may provide important data for resolving conflicting results based on different diagnostic techniques and establishing a fundamental theoretical basis for future research on this equid retrovirus.

## 2. Materials and Methods

### 2.1. Ethics Statement

The experiments were approved by the Institutional Animal Care and Use Committees from the School of Veterinary Medicine and Animal Science (protocol number 195/2012-CEUA), São Paulo State University (UNESP), São Paulo, Brazil.

### 2.2. Sample Collection and RNA Purification

Plasma from two horses (BRA1 and BRA2) from Poconé, MT, Brazil, was collected for this study, both were positive for EAIV according to AGID [21] and ELISA [22] tests. BRA1 was an asymptomatic viral carrier, and BRA2 presented oedema of low parts, high fever, and hypochlorous mucous membranes. Both animal samples were positive by semi-nested PCR with reverse transcription analysis [23], confirming the presence of viral RNA. Plasma was previously centrifuged at 8000× g for 10 min, and 200 µL of the pellet was used for RNA extraction with TRIzol LS Reagent (Invitrogen, Carlsbad, CA, USA) according to the manufacturer’s instructions. RNA samples were quantified using the commercial fluorometer Qubit 2.0 (Invitrogen) with an RNA assay kit (Invitrogen), and 260/280 and 260/230 were measured with a NanoDrop™ 1000 Spectrophotometer (Thermo Fisher Scientific, Waltham, MA, USA). Samples were then treated with DNase I (Sigma-Aldrich, Saint Louis, MO, USA), according to the manufacturer’s instructions.

### 2.3. Library Preparation and Next-Generation Sequencing

The BRA1 dsDNA library was synthesized from total RNA with a strand-specific RNA library prep kit (Agilent Technologies, Santa Clara, CA, USA) following the manufacturer’s recommendations and quantified with an Illumina library quantification kit (KAPA Biosystems, Wilmington, MA, USA). The qPCR product generated was fractionated by 1.5% agarose horizontal gel electrophoresis.

The BRA2 library was prepared with a Nextera XT DNA library preparation kit (Illumina Inc., San Diego, CA, USA) and quantified using a KAPA library quantification kit for the Illumina Sequencing platform (KAPA Biosystems) according to a previously described protocol [24].

The BRA1 library was sequenced with the NextSeq System (Illumina) using a NextSeq 550 System high-output kit (1 × 75 cycles). The BRA2 library was sequenced using a NextSeq mid-output kit (2 × 150 cycles) (Illumina).

### 2.4. Bioinformatics Analysis

Initially, raw data (reads) were evaluated using the program Trim_Galore v.0.6.5 [25] to remove possible adapters. The data were then filtered and trimmed for sequence quality and size (<50 bp) using the PRINSEQ-lite algorithm [26]. Afterwards, de novo analysis was performed with the IDBA-UD v.1.1.3 [27] and SPAdes v.3.13.1 programs [28] to assemble the reads into contigs. Comparison with the non-redundant protein database was carried out by BlastX implemented in the DIAMOND tool v.0.9.26 [29], with an E-value of 0.00001 [30]. Data visualization using the Krona tool [31] allowed intuitive exploration of relative abundances and confidences within the complex hierarchies of taxonomic classifications of the generated contig sets. Geneious R8 v.8.1.9 [32] was used to complete the bioinformatics analysis by mapping the contigs and high-quality reads to a reference genome. EIAV genome sequences Liaoning (AF327877), Wyoming (AF033820), Miyazaki 2011-A (JX003263), IRE F2 (JX480631), Devon 2010 (MH580897), and Cornwall 2012 (MH580898) and two incomplete sequences, ITA DE (KM247554) and ITA SA (KM247555), were aligned with the NGS reads. Alignments and phylogenetic trees were built with MEGA 7 software [33]. Sequences were aligned by the MUSCLE algorithm v.3.8.31, and trees were constructed using the maximum-likelihood algorithm. The Tamura-Nei model was employed to stipulate distances with bootstrapping of 1000 replicates.

### 2.5. Primer Design and Sanger Sequencing

Primers were designed for different regions of the EIAV genome, flanking products from 800 to 1300 bp, as based on a consensus sequence obtained by massive sequencing to cover possible gaps and validate the obtained consensus sequence. The primers were designed using Primer 3 software [34] and analyzed by OligoAnalyzer 3.1, available online on the Integrated DNA Technologies-IDT site (https://www.idtdna.com/calc/analyzer), and by Primer-BLAST software (www.ncbi.nlm.nih.gov) (Table 1).

The PCR products were amplified in a reaction with 2 × GoTaq Green Master Mix (Promega, Madison, WI, USA), 0.3 mM of each primer, 5 µL of sample, and 25 µL of nuclease-free water q.s.p. The conditions of the first PCR cycles consisted of an initial denaturation for 10 min at 95 °C, 5 repeated cycles of 30 s at 95 °C, 30 s at 60 °C, and 2 min at 72 °C, 5 repeated cycles of 30 s at 95 °C, 30 s at 55 °C, and 2 min at 72 °C, and 30 repeated cycles of 30 s at 95 °C, 30 s at 50 °C, and 2 min at 72 °C, followed by 10 min of final extension at 72 °C. Amplification was analyzed by 1.5% horizontal agarose gel electrophoresis and purified for Sanger sequencing. Internal primers were designed to enhance Sanger sequencing for large products. The amplified product of the *env* gene region (nt 5112 to 8000; see Table 1) could not be sequenced with PCR purification alone, thus, the fragment was inserted into the pGEM-T Easy Vector (Promega) and transformed into *Escherichia coli* DH5-α following the manufacturer’s protocol. For PCR product sequencing, we used BigDye Terminator v3.1 cycle sequencing (Life Technologies), with a reaction per primer following the manufacturer’s directions. Capillary electrophoresis and analysis were performed using an ABI 3500 automatic sequencing system (Life Technologies).

### 2.6. Variability Analysis

A search for variability and single-nucleotide polymorphisms (SNPs) was performed using all the aligned reads for the final consensus BRA1 sequence obtained, which presented an average coverage greater than 1000 reads. As the estimated sequencing error rate with the Illumina platform is approximately 2%, we stipulated a minimum coverage of 10 and a minimum variance frequency of 0.2 for analysis using Geneious R8 v. 8.1.9 program tools.

### 2.7. Molecular Modelling

A molecular model of the *gag* protein p26 was built to verify possible variations of epitopes, because this protein is used for serological diagnosis (AGID). This molecular model was based on the 2.7 Å resolution-crystallographic structure of p26 (1EIA) from a previous X-ray diffraction experiment [35]. Linear p26 amino acid sequences from BRA1 and BRA2 were used to search for homology sequences for threading using the HHpred server (https://toolkit.tuebingen.mpg.de/#/tools/hhpred) [36]. Modeller 9.15 [37] was applied to generate the p26 models, and the best model was chosen based on stereochemistry parameters assayed on the MOLProbity server (http://molprobity.biochem.duke.edu/) [38]. Previously described epitope sequences [39,40,41] were compared by structure alignment using the CHIMERA program [42].

## 3. Results

### 3.1. Complete Sequence of Brazilian EIAV

Next-generation sequencing using the Illumina platform was performed with two RNA samples obtained from the plasma samples of two naturally infected horses. The sequencing of equine 1 (BRA1) yielded more reads than did the sequencing of equine 2 (BRA2) (Table 2). The sequences were aligned using six complete EIAV genomes as references: Liaoning (AF327877), Wyoming (AF033820), Miyazaki2011-A (JX003263), IRE F2 (JX480631), Devon 2010 (MH580897), Cornwall 2012 (MH580898), and two incomplete sequences, ITA DE (KM247554) and ITA SA (KM247555), obtained from field animals. The overall alignment presented some gaps in different consensus regions of the viral genome, especially in the *env* gene.

In order to cover the remaining regions and to obtain the complete genomic sequence, primers were designed based on the partial consensus BRA1 and BRA2 sequences and their sequenced products. The PCR product corresponding to the *env* gene did not show multiple bands by gel electrophoresis, however, the results indicated that more than one product had been amplified. In this case, cloning of the products was performed, and six individual colonies for BRA1 and five colonies for BRA2 were sequenced.

The sequences obtained by Sanger were incorporated into the map for reference analysis with each complete genome already described. Such sequences acted as a guide for the highly divergent regions of the original sequence and helped with genome coverage. The consensus sequences ultimately obtained for BRA1 and BRA2 presented 8016 and 7968 bases, respectively, from R to R (LTR), ending with a poly A tail, a characteristic typical of retroviruses. The consensus sequences were annotated with prediction ORFs based on other genome annotations.

**Figure 1:**
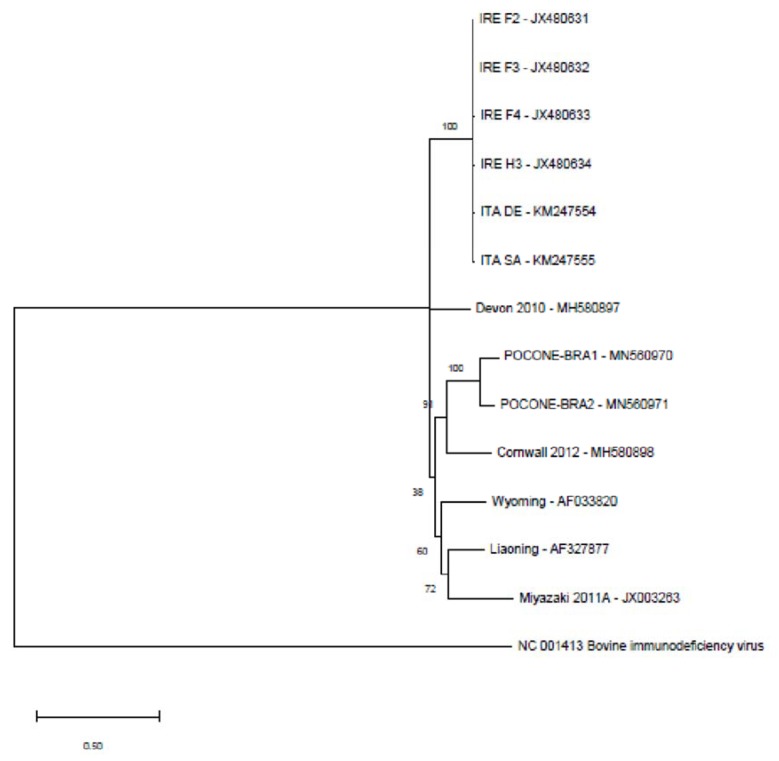
Cladogram involving equine infectious anemia virus (EIAV) complete genome sequences and BRA1 and BRA2 sequences obtained from the Pantanal region using the maximum-likelihood method. The following sequences were used: EIAV Miyazaki 2011A (Japan); EIAV BRA1 and EIAV BRA2 (Brazil); EIAV Liaoning (China); EIAV Wyoming (USA); EIAV ITA SA and EIAV ITA DE (Italy); EIAV IRE F2, EIAV IRE F3, EIAV IRE F4 and EIAV IRE H3 (Ireland); EIAV Cornwall 2012 and EIAV Devon 2010 (England); bovine Immunodeficiency virus (outgroup—NC_001413.1).

Multiple alignments of the complete sequences were performed to generate a phylogenetic tree based on the maximum-likelihood method. Identities between the described genomes and the Brazilian sequences are provided in Table 3. European sequences exhibit higher identity, with degrees of identity varying between 98.22% and 99.42%. However, genomes from different world regions show identity of approximately 74% to 79%. In contrast, the Brazilian sequences (BRA1 and BRA2) present an identity of 88.56%. The phylogenetic tree showed clades containing sequences from mixed geographic locations: England/Brazil, Europe, Asia/USA (Figure 1).

### 3.2. Polymorphisms in the EIAV Genome

**Figure 2:**
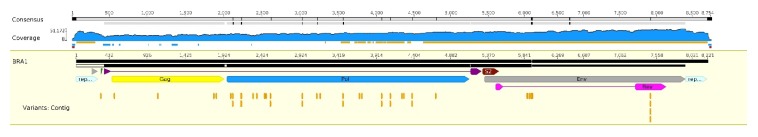
Representative genome of EIAV BRA1. Genome annotation shows the variant distribution, marked below in yellow. Coverage is represented in blue, and identity is represented in dark yellow.

Genetic variation between the viral genomes comprised SNPs, insertions, and deletions. Sufficient coverage for variation analysis was only achieved for the BRA1 sample (coverage above 1000 reads). In total, 33 SNPs were identified in the BRA1 reads, with 19 causing amino acid changes (Supplemental Figure 1). The highest number of SNPs was found for the *pol* gene (21), followed by *env* (7), and *gag* (4). Only one SNP was located in the U5 LTR (Figure 2). No insertions or deletions passed the cut-off frequency of 0.25.

Sanger sequences of *env* were assembled to compare variations in this gene. Sequences from the same equine show insertions and deletions from 3 to 24 nucleotides (1 to 6 amino acids) and nucleotide/amino acid substitutions (Supplementary Figure S1). It was not possible to sequence the total *env* gene by Sanger sequencing using only one PCR fragment, therefore, more than one fragment was necessary to cover the whole gene. However, due to the presence of different PCR fragments obtained from different quasispecies present in the BRA1 sample, we were not able to define the precise sequence of each specific *env* gene.

### 3.3. LTR and Viral Genes

Unlike proviral genomes, the genomes of Brazilian EIAV do not carry a complete LTR. The BRA1 sequence consisted of 192 bases for U3, 76 bases for R, and 39 for U5. The BRA2 sequence consisted of 191 bases for U3, 78 bases for R, and 40 bases for U5. The sequences of 5′UTR and 3′UTR were compared to confirm that the R region was present in both LTRs. In total, the complete BRA1 LTR and BRA2 LTR (U3 + R + U5) sequences comprised 307 bases and 309 bases, respectively. The Brazilian LTR sequences were aligned with EIAV LTRs from field virus full-genome sequences and Chinese sequences [43]. BRA1 and BRA2 LTR showed 92.6% identity with one another, with variation between 61.91 and 72.92% compared with the foreign sequences. Most of the variation was found to be concentrated in the U3 region, with the initiation site of transcription changes from GGG/A to AGA in Brazilian sequences. Additional conserved motifs were identified: a methylated DNA-binding protein site (MDBP, also known as EF-C or EP) with variations (BRA1 = GTTGCTAGGCAAC, BRA2 = GATGATAAGCAAC), an absent PEA1/AP-1 site in both LTRs, only two PU.1 binding sites (one site in a Wyoming strain and three in the other sequences), and a TATA box and transactivation response element (TAR) without alterations (Supplementary Figure S2). LTR phylogenetic analysis was also performed for comparison with the full genome. The results from this phylogenetic analysis indicated that sequences from the same geographic regions form monophyletic groups, as also observed for BRA1 and BRA2 sequences, which were separated in an exclusive clade (Supplementary Figure S6).

The *gag* genes of both BRA1 and BRA2 consisted of 1464 nucleotides that encode 487 amino acids of the *gag* polyprotein (124 in p15, 232 in p26, 79 in p11, and 52 in p9). Brazilian *gag* sequences (BRA1 and BRA2) showed identity of 91.12%, but an identity of between 77 and 79% in comparison to other sequences. However, there were more amino acid variations between the Brazilian sequences (7 amino acids) in relation to the European sequences (1 amino acid), which displayed a high degree of identity (approximately 99%). The leucine-rich nuclear export signal was conserved in all p15 proteins, and the major homology region (MHR) of p26 presented the same sequence as that of the Liaoning strain (IRQGPKEPYPEFVDRLLSQI). The first p11 zinc-binding domain was conserved and the second domain had a Q/E substitution, as observed in the Miyazaki strain. The YPDL late domain in p9 showed no variations (Supplementary Figure S3).

The polyprotein *pol*, the most conserved among retroviruses, exhibited an overall identity between 79.8% and 99.8% when considering all analyzed sequences. The Brazilian *pol* sequences consisted of 3414 nucleotides encoding a polypeptide chain of 1137 amino acids, which was cleaved into five proteins: protease (PR), reverse transcriptase (RT), RNase H (RN), dUTPase (DU), and integrase (IN). The BRA1 and BRA2 sequences showed 91.7% identity. The protease residues that form the substrate binding site, the DTG catalytic motif, and the active site flap were well conserved among all field and Brazilian strains (Supplementary Figure S4). Only Miyazaki and Wyoming presented an alteration of valine to leucine or isoleucine in the active site flap (both nonpolar amino acids). The RT motifs described for HIV were also conserved in BRA1 and BRA2 (DIGD and DD) [41]. RN contained four invariant residues and a glycine-rich motif conserved in all sequences. Of the five conserved motifs described for DU, the Brazilian sequences showed alterations in residues of M5 (BRA1 = RGEEGFGSTG; BRA2 = RGEKGFGSTG), conserved residues of M3, which are responsible for uracil ring binding (N67, G69, Y75, Q80, and I82), and conservation of the residue D72, which also occurs in all of the other strains analyzed. Regarding IN, the zinc-binding HHCC motif (H12, H16, C40, C43), hydrophobic core residues (I5, A8, L22, A33, and I36), and three central catalytic core residues (D64, D116, and E152) were not altered.

The EIAV *env* sequence, which encodes the surface protein (gp90) and transmembrane protein (gp45), consisted of 2613 nucleotides encoding 872 amino acids for BRA1 and 2568 nucleotides encoding 855 amino acids for BRA2. This gene is the most variable EIAV gene, with 72.83% identity between BRA1 and BRA2 sequences and at least 57.33% identity with other field viruses. gp90 protein variation was identified in 8 hypervariable regions (V1 to V8), with several mutations (Supplementary Figure S5). In fact, only the first 12 amino acids and cysteine residues of gp90 were conserved among all sequences analyzed. More variations between Brazilian sequences were also observed compared to European sequences. In contrast, the gp45 protein transmembrane domain carried one amino acid alteration between BRA1 and BRA2.

Phylogenetic trees were built based on the alignment of each analyzed gene: *gag*, *env*, and *pol* (Supplementary Figure S6). Due to the high variability of *env*, two phylogenetic trees were built for this gene based on nucleotide and amino acid alignments, however, the results indicated a similar grouping for each. The phylogenetic analysis showed different clusters for strains in each EIAV gene, and these results did not corroborate with the full-genome tree.

### 3.4. Gag Variation and p26 Molecular Modelling

**Figure 3:**
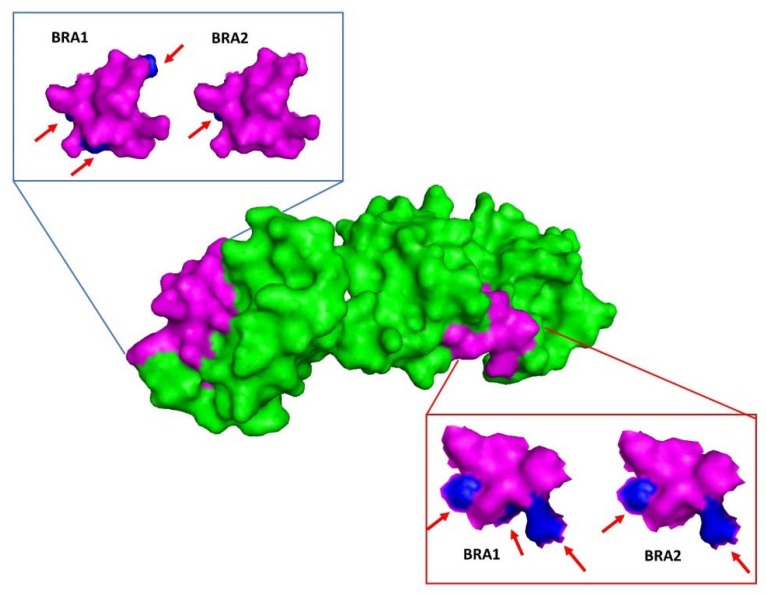
Molecular modelling of BRA1 and BRA2 p26 using a p26 crystallographic structure (PDB code 1EIA) with Modeller software. Epitopes with amino acid alterations were compared in both models, showing surface residue alterations. Blue arrows point to amino acid alteration compared to the reference.

Brazilian p26 is 232 amino acids long, with alterations between BRA1 and BRA2 (Table 4). Analysis of these alterations indicated no alteration in the physical and chemical characteristics of the modified residues. Models of p26 BRA1 and BRA2 were analyzed with regard to epitope structural analysis. All altered residues in the epitopes were located on the protein surface (Figure 3).

## 4. Discussion

This report is the first to describe a complete EIAV genome from naturally infected Brazilian horses (field samples). Partial sequence of the 5′ LTR/*tat* fragment was previously obtained from equids in the Pantanal region [23], and nucleotide sequences showed similarity from 93.5% to 100% with our sequences. Partial sequences of the Brazilian EIAV *gag* gene from Bahia are already available at NCBI (accession numbers: KC213776–KC213790) [44]. Bahia is a Brazilian state considered a low-risk area for EIA, thus, euthanasia of EIA-positive animals is mandatory, as it is for other low-risk areas of the country. Comparison of Bahia’s sequences and BRA1/BRA2 revealed some nucleotide substitutions, indicating that the circling virus in Pantanal is different from the current virus circulating in a controlled EIA area in Brazil (Supplementary Figure S7). High similarities were found among EIAV isolates from Bahia and Pantanal (present study) with European and North American strains [44]. A phylogeography to investigate the spatiotemporal dynamics of EIAV revealed Hungary as the origin of EIAV and described a migration route from Europe to the USA, then diversified and spread throughout Brazil [45]. We can observe a diversification or different origin of Brazilian strains by comparing Bahia and Pantanal *gag* sequences: Bahia’s sequences are closer to the USA sequence, as described in the origin article [44], and BRA1/BRA2 are clustered with Italy/Ireland sequences (Supplementary Figure S8). The restricted circulation of positive equids in Pantanal may have contributed to these results.

Full-length field sequences from other countries (except the Wyoming strain) have been described based on proviral DNA samples, but not genomic RNA. Although the EIAV_LIA_, EIAV_IRE_, EIAV_MIY_, and EIAV_WY_ strains were sequenced using the Sanger method, NGS was employed to obtain the England sequences [10], and a partial sequence of EIAV_ITA_ was obtained from amplification of 7841 bp of EIAV [11]. Both animals used in this study exhibited viremia at sample collection. This fact was confirmed by PCR. Usually, positive samples amplify only in semi-nested PCR reactions, but amplification occurred for both samples in the first PCR reaction (data not shown). This high viral level allowed good results in NGS library preparation from mRNA without previous amplification. Complete field sequences described in the USA, Japan, China, Ireland, England, and partial Italian sequences were compared to the new Brazilian sequences obtained in this study. We did not succeed in improving the RNA purification with the different kits and protocols tested, prior enzymatic treatment for host DNA/RNA degradation, serial centrifugation cycles to obtain isolated virus fractions, cDNA synthesis with oligo dT primer, and/or viral capture with specific primers (data not shown). Moreover, it was not possible to complete the viral sequence only using the reads obtained by NGS. To complete the gaps, PCR with primers based on BRA1 and BRA2 incomplete sequences were used to amplify regions of the viral genome for Sanger sequencing. The *env* gene product could not be sequenced by Sanger sequencing, and a cloning strategy was implemented.

After cloning, different viral *env* sequences were found for the same equine sample, and this level of variation is common among lentiviruses, in which the viral RT is error-prone because it does not possess a proofreading mechanism. Therefore, this property leads to a gradual accumulation of different populations of viruses that differ slightly in their RNA sequence, providing diversity for natural selection [4]. The *env* gene is the most variable in lentiviruses, with gp90 mutations responsible for the generation of immune system escape mutants [9]. Due to this high variability, it is difficult to build a reference map using alignment algorithms that provides a reliable consensus for this genomic region. In fact, the *env* gene present low identity among the aligned reads (Figure 2) and the presence of SNPs (NGS data), insertions, and deletions (Sanger data) and V1 to V8 hypervariable regions with many alterations when compared to reference sequences [46].

Studies of EIAV genomic variation during persistent infection in experimentally infected equids have shown that febrile peaks and re-emergence of clinical signs are clearly associated with modified antigenic properties of the virus envelope protein. The predominant site of EIAV variation during persistent infection is gp90 [47], variations of this protein were identified in eight hypervariable regions (V1 to V8). These regions possess distinct signatures, especially in the V3 and V4 domains, which allow the differentiation of quasispecies [48]. In the BRA1 V3 region, 6 SNPs (G5881A; C5885G; A5912T; C5929A; A5941C; C5943T) as well as some nucleotide alterations, insertions, and deletions were found in BRA1 clones (Supplementary Figure S1). V4 is a shorter region than V3. Although no SNPs were found, an insertion was present in the sequenced BRA2 clones (Supplementary Figure S1).

The presence of two distinct signatures in the *env* sequence indicated that the equines were infected by different viral quasispecies. This fact is expected for an endemic region, where the host immune system response associated with natural selection is not the only cause of quasispecies emergence, as the proximity of several infected equines living in the same environment for many years allows the exchange of circulating viruses, either by vectors or human activity [49]. Nevertheless, for the *env* gene, the glycoprotein gp45 (transmembrane protein) is a smaller protein with less variation than gp90. For this reason, gp45 is used in some ELISA tests as a complementary antigen to p26 [2,50]. In fact, the Brazilian sequences of gp45 presented regions with identity of more than ten amino acids to those observed in other strains with only one amino acid (A7480G), a region shared with the *rev* gene, and an insertion of three nucleotides (one amino acid). Nonetheless, the *po*l gene is described as a conserved sequence because the proteins associated with this gene are essential for completing viral replication. In fact, for BRA1, this gene was shown to be more synonymous with SNPs than the other genes, resulting in a high degree of conservation for *pol* proteins.

The *gag* polyprotein produces four proteins (matrix p15, capsid p26, nucleocapsid p11, and the late protein p9) by proteolytic cleavage. Among these proteins, p26 is particularly important for generating the immune response detected by AGID, which is considered the gold standard diagnostic method for EIA [13]. In addition to having high antigenicity, p26 is used for EIA diagnosis due to its high conservation in different strains. Moreover, the PCR diagnostic tests based suggested by OIE are based on detection of this gene. Therefore, alterations in the p26 nucleotide sequence might result in epitope structural modifications, thereby influencing the efficiency of serological diagnosis. Indeed, serologically silent equines have been observed in several regions of the world [51]. In our study, both Brazilian p26 proteins displayed changes in surface amino acids in the described epitopes [39,40,41] (Figure 3). These results suggest that more studies related to *gag* gene characterization may contribute to better detection of EIAV infection and disease control in an endemic area (at least in the Pantanal region), where adaptation by the virus to their hosts allows the persistence of infected animals for years.

Finally, comparison of EIAV gene sequences between the novel Brazilian sequences and other sequences available provided important information about the extent of variation between viral strains from distinct geographic regions. Indeed, EIAV sequences can be separated into local groups, indicating that this virus can be grouped in a manner similar to HIV, which is divided into groups and subtypes according to origin and phylogeny [52]. Although not characterized for EIAV, homologous recombination likely occurs when a cell is co-infected with two different but related strains [53]. Naturally occurring recombinant EIAV strains can be found in infected equids in endemic regions where multiple genotypic variants co-circulate. As there are not enough EIAV sequences to perform similar classification or recombination analysis, the present study represents a significant step in improving our understanding of molecular conservation and variation in this important equine lentivirus.

## 5. Conclusions

Here, complete genomic sequences of Brazilian EIAV (BRA1 and BRA2) from an area at high risk of EIA transmission area are described. The NGS strategy developed was able to generate sequences without pre-amplification of the RNA equine sample. Based on sequence alignments and similarity plot analysis, BRA1 and BRA2 are not closely related to any other strain reported to date, forming a separate monophyletic group that can be classified as a novel EIAV strain. Based on *env* gene analysis, different viral quasispecies were also observed in both equines. Studies on variations in the EIAV genome can help elucidate the viral complexity and evolutionary mechanisms of EIAV in natural and persistent infection in areas of high viral circulation.

## Figures and Tables

**Table 1:** Primers for equine infectious anemia virus (EIAV) genome regions. Genome positions are according to MN560970.

Primer	Sequence 5′-3′	Genome Position	Product Size
AIE_GEN F 463-482	CCA GAG CAC AGG ARG ACA GG	463-482	
AIE_GEN R 926-906 *	CCT CTG GGK GTT AAG GGT CGG	926-906	889 bp
AIE_GEN R 1352-1333	TAA GGC TCT TTK GGS CCT TG	1352-1333	
AIE_GEN F 1333-1352	CAA GGS CCM AAA GAG CCT TA	1333-1352	
AIE_GEN R 1963-1943 *	TTA CTC CCA CAA ACT GCT CAG	1963-1943	940 bp
AIE_GEN R 2273-2254	GCC ATT ACC AAT TGT GCC CC	2273-2254	
AIE_GEN F 3878-3897	GCC AGG TCA CAA GGG CAT AT	3878-3897	
AIE_GEN R 4540-4521 *	CCT GCA GGT CCA GAT CCY TG	4540-4521	1096 bp
AIE_GEN R 4974-4955	CCA TGG TGT TTG KCC YCC CA	4974-4955	
AIE_GEN F 5112-5132	GGG TGA TGG TGC TGT AGT GGT	5112-5132	
AIE_GEN F 5934-5959 *	GTG TAC AGA TAG TGA TCA TTG TCA AG	5934-5959	1637 bp
AIE_GEN R 6749-6727	GCC CGA GAA GTA ACA GGA AAA GG	6749-6727	
AIE_GEN F 6823-6842	GCT ATT GCT GCT AGT GCY AC	6823-6842	1177 bp
AIE_GEN R 8000-7981	AGA TGT AGC TGG ATT TAR CG	8000-7981	
AIE_GEN F 7751-7770	CAA AGC GAA GGA GGA AAC AT	7751-7770	526 bp
AIE_GEN R 8277-8258	GGG ACT CAG ACC GCA GAA TC	8277-8258	

* Used as an inner primer for Sanger sequencing.

**Table 2:** Summary statistics for the two equine infectious anemia virus sequenced genomes.

Samples	Raw Reads	Reads at Least Q30	Mapped Reads	Mean Coverage
BRA1	18,371,626	92.7%	422,676	3969
BRA2	10,374,696	71.2%	6927	78.3

**Table 3:** The identity matrix with EIAV genomes shows the percentage of bases/residues that are identical.

	Miyazaki	BRA1	BRA2	Cornwall	Wyoming	Devon	Liaoning	IRE H3	IRE F4	IRE F2	IRE F3	ITA SA	ITA DE
Miyazaki - JX003263													
BRA1 - MN560970	74.963												
BRA2 - MN560971	74.944	88.507											
Cornwall 2012 - MH580898	72.507	75.171	75.440										
Wyoming - AF033820	75.156	76.601	76.614	74.829									
Devon 2010 - MH580897	75.897	76.500	77.322	75.255	77.490								
Liaoning - AF327877	76.531	76.751	77.491	75.115	77.860	78.906							
IRE H3 - JX480634	75.568	77.021	76.741	75.044	78.189	78.855	79.018						
IRE F4 - JX480633	75.520	77.058	76.741	74.994	78.196	78.951	79.066	99.190					
IRE F2 - JX480631	75.348	77.042	77.015	75.224	78.393	79.080	79.297	98.937	99.118				
IRE F3 - JX480632	75.612	77.005	76.741	75.053	78.149	78.934	78.990	99.057	99.299	99.420			
ITA SA - KM247555	75.455	76.756	76.823	75.409	78.338	79.128	79.252	98.512	98.792	99.197	98.983		
ITA DE - KM247554	75.253	76.630	76.798	75.282	78.288	79.014	79.176	98.461	98.665	99.133	98.944	99.324	

**Table 4:** Previously described epitope alterations for each p26 sequence obtained. Alterations are marked in red.

Reference	Epitope	BRA1	BRA2
[35]	^73^NLDKIAEE^80^	^73^LLDKMAED^80^	^73^LLDKIAED^80^
[35]	^199^KNAMRHLRPEDTLEEKMYAC^218^	^199^RNAMRHLRPEDSLEEKLYAC^218^	^199^KNAMRHLRPEDTLEEKLYAC^218^
[33]	^158^KEPYPEFVDRLLSQI^172^	^158^KEPYPEFVDRLLSQI^172^	^158^KEPYPEFVDRLLSQI^172^
[34]	^200^NAMRHL^205^	^200^NAMRHL^205^	^200^NAMRHL^205^
[34]	^215^MYACRD^220^	^215^LYACRD^220^	^215^LYACRD^220^

## Supplementary Materials

The following are available online at https://www.mdpi.com/1999-4915/12/2/207/s1. Table S1: Table of SNPs for BRA1, Figure S1: Alignment of *env* clone sequences of BRA1 and BRA2 based on Sanger sequencing, Figure S2: Alignment of nucleotide sequences in the EIAV LTR, Figure S3: Alignment of the EIAV Gag polyprotein, Figure S4: Alignment of the EIAV Pol polyprotein, Figure S5: Alignment of the EIAV *env* region, Figure S6: Phylogenetic trees of Brazilian field sequences and sequences from abroad, Figure S7: Multiple alignment of *gag* sequences from Bahia (Brazil) and BRA1 and BRA2 from Pantanal (Brazil).
