# Supplementary material for: High Genomic Variability in Equine Infectious Anemia Virus Obtained from Naturally Infected Horses in Pantanal, Brazil: An Endemic Region Case
Source: Viruses. 2020 Feb 12;12(2):207. doi: 10.3390/v12020207 (PMC7077297; doi:10.3390/v12020207)

Supplementary Table S1: Anoted SNPs from BRA1. Genome position number is given as BRA1 consensus reference.

Genome position	Change	Coverage	Variant Frequency	Variant P-Value	Amino Acid Change	Codon Change	Protein Effect	CDS
**327**	T -> A	201	25.90%	1.00E-123				LTR
**502**	A -> G	162	28.40%	8.40E-112	K -> R	AAG -> AGG	Substitution	Gag
**1,067**	G -> A	123	29.30%	3.80E-90		AAG -> AAA	None	Gag
**1,799**	G -> A	241	37.30%	2.50E-239		GAG -> GAA	None	Gag
**1,830**	A -> G	263	34.20%	1.10E-216	N -> D	AAC -> GAC	Substitution	Gag
**2,024**	T -> C	471	37.80%	0.00E+00		TTA -> CTA	None	Pol
**2,046**	A -> G	387	45.00%	0.00E+00	K -> R	AAG -> AGG	Substitution	Pol
**2,155**	G -> T	347	39.80%	0.00E+00	L -> F	TTG -> TTT	Substitution	Pol
**2,157**	A -> G	329	48.60%	0.00E+00	K -> R	AAG -> AGG	Substitution	Pol
**2,308**	T -> C	125	27.20%	3.40E-86		ATT -> ATC	None	Pol
**2,364**	G -> A	191	34.60%	6.80E-173	R -> K	AGA -> AAA	Substitution	Pol
**2,462**	A -> T	649	36.70%	0.00E+00	I -> L	ATA -> TTA	Substitution	Pol
**2,478**	C -> T	423	33.60%	0.00E+00	A -> V	GCT -> GTT	Substitution	Pol
**2,539**	A -> G	433	46.70%	0.00E+00		GGA -> GGG	None	Pol
**2,952**	A -> G	465	40.90%	0.00E+00	N -> S	AAT -> AGT	Substitution	Pol
**3,144**	A -> G	399	46.60%	0.00E+00	K -> R	AAA -> AGA	Substitution	Pol
**3,184**	G -> A	463	25.10%	1.90E-251		CAG -> CAA	None	Pol
**3,482**	A -> G	1,307	48.90%	0.00E+00	I -> V	ATA -> GTA	Substitution	Pol
**3,699**	G -> A	1,149	28.00%	0.00E+00	R -> K	AGG -> AAG	Substitution	Pol
**3,727**	C -> T	1,131	34.80%	0.00E+00		GAC -> GAT	None	Pol
**3,985**	C -> T	1,226	50.70%	0.00E+00		TAC -> TAT	None	Pol
**4,099**	A -> G	1,753	47.70%	0.00E+00		GGA -> GGG	None	Pol
**4,249**	G -> A	1,709	26.90%	0.00E+00		CAG -> CAA	None	Pol
**4,273**	T -> C	1,589	35.40%	0.00E+00		TCT -> TCC	None	Pol
**4,381**	A -> G	871	48.90%	0.00E+00		GCA -> GCG	None	Pol
**4,693**	A -> G	2,881	50.20%	0.00E+00		CAA -> CAG	None	Pol
**5,881**	G -> A	2,164	32.20%	0.00E+00	R -> K	AGA -> AAA	Substitution	Env
**5,885**	C -> G	2,104	32.70%	0.00E+00	N -> K	AAC -> AAG	Substitution	Env
**5,912**	A -> T	1,847	32.00%	0.00E+00	K -> N	AAA -> AAT	Substitution	Env
**5,929**	C -> A	2,105	97.10%	0.00E+00	T -> N	ACC -> AAC	Substitution	Env
**5,941**	A -> C	2,259	98.80%	0.00E+00	H -> P	CAT -> CCT	Substitution	Env
**5,943**	C -> T	2,283	99.20%	0.00E+00	H -> Y	CAT -> TAT	Substitution	Env
**7,480**	A -> G	16,497	54.30%	0.00E+00	N -> S	AAT -> AGT	Substitution	Env/Rev

Supplementary Figure S1: Alignment of env clone sequences of BRA1 and BRA2. The BRA1 sequence was used as reference to the alignment. Inclusions and deletions are marked in yellow. Hipervariable region V3 is highlighted in green and V4 in pink.

```
                    1        10        20        30        40        50        60
                    |        |         |         |         |         |         |
BRA1                ATGGTCAGCATCGCGTTCTATGGGGGAATCCCAGGGGGAATCTCGACCCCTATTATTCAA
BRA1 Sequence 3     ATGGTCAGCATCGCGTTCTATGGGGGAATCCCAGGGGGAATCTCGACCCCTATTATTCAA
BRA2 sequence 4.1   ------------------------------------------------------------
BRA1 Sequence 1     ------------------------------------------------------------
BRA2 sequence 5.1   ------------------------------------------------------------
BRA1 Sequence 2     ------------------------------------------------------------
BRA2 sequence 2.1   ------------------------------------------------------------
BRA2 sequence 2.2   ------------------------------------------------------------
BRA2 sequence 4.2   ------------------------------------------------------------
BRA2 sequence 5.2   ------------------------------------------------------------
BRA2 sequence 3.2   ------------------------------------------------------------
BRA2 sequence 6.2   ------------------------------------------------------------
BRA1 Sequence 1     ------------------------------------------------------------
BRA2 sequence 1.1   ------------------------------------------------------------
BRA1 Sequence 4     ------------------------------------------------------------
BRA2 sequence 3.1   ------------------------------------------------------------
BRA1 Sequence 2     ------------------------------------------------------------

BRA1                TCTTCAGAAGAAGCTAGTCACACCAAGGGGGATACAATATTTAAACCCTATTGTTCTGAT
BRA1 Sequence 3     TCTTCAGAAGAAGCTAGTCACACCAAGGGGGATACAATATTTAAACCCTATTGTTCTGAT
BRA2 sequence 4.1   ------------------------------------------------------------
BRA1 Sequence 1     ------------------------------------------------------------
BRA2 sequence 5.1   ------------------------------------------------------------
BRA1 Sequence 2     ------------------------------------------------------------
BRA2 sequence 2.1   ------------------------------------------------------------
BRA2 sequence 2.2   ------------------------------------------------------------
BRA2 sequence 4.2   ------------------------------------------------------------
BRA2 sequence 5.2   ------------------------------------------------------------
BRA2 sequence 3.2   ------------------------------------------------------------
BRA2 sequence 6.2   ------------------------------------------------------------
BRA1 Sequence 1     ------------------------------------------------------------
BRA2 sequence 1.1   ------------------------------------------------------------
BRA1 Sequence 4     ------------------------------------------------------------
BRA2 sequence 3.1   ------------------------------------------------------------
BRA1 Sequence 2     ------------------------------------------------------------

BRA1                GAAAAAAGTAACAGGAATAAAACAATGGCGGAAGAAAGAGATTCAAGAGAACGAAGAGAA
BRA1 Sequence 3     GAAAAAAGTAACAGGAATAAAACAATGGCGGAAGAAAGAGATTCAAGAGAACGAAGAGAA
BRA2 sequence 4.1   ------------------------------------------------------------
BRA1 Sequence 1     ------------------------------------------------------------
BRA2 sequence 5.1   ------------------------------------------------------------
BRA1 Sequence 2     ------------------------------------------------------------
BRA2 sequence 2.1   ------------------------------------------------------------
BRA2 sequence 2.2   ------------------------------------------------------------
BRA2 sequence 4.2   ------------------------------------------------------------
BRA2 sequence 5.2   ------------------------------------------------------------
BRA2 sequence 3.2   ------------------------------------------------------------
BRA2 sequence 6.2   ------------------------------------------------------------
BRA1 Sequence 1     ------------------------------------------------------------
BRA2 sequence 1.1   ------------------------------------------------------------
BRA1 Sequence 4     ------------------------------------------------------------
BRA2 sequence 3.1   ------------------------------------------------------------
BRA1 Sequence 2     ------------------------------------------------------------

BRA1                GCGTACCTGAAAGAAGATGCAGAAGAAAATGCAAAGAAGAGAAACAATGACTGGTGGAAA
BRA1 Sequence 3     GCGTACCTGAAAGAAGATGCAGAAGAAAATCCAAAGAAGAGAAACAATGACTGGTGGAAA
BRA2 sequence 4.1   ------------------------------------------------------------
BRA1 Sequence 1     ------------------------------------------------------------
BRA2 sequence 5.1   ------------------------------------------------------------
BRA1 Sequence 2     ------------------------------------------------------------
BRA2 sequence 2.1   ------------------------------------------------------------
BRA2 sequence 2.2   ------------------------------------------------------------
BRA2 sequence 4.2   ------------------------------------------------------------
BRA2 sequence 5.2   ------------------------------------------------------------
BRA2 sequence 3.2   ------------------------------------------------------------
BRA2 sequence 6.2   ------------------------------------------------------------
```

```
BRA1 Sequence 1     ------------------------------------------------------------
BRA2 sequence 1.1   ------------------------------------------------------------
BRA1 Sequence 4     ------------------------------------------------------------
BRA2 sequence 3.1   ------------------------------------------------------------
BRA1 Sequence 2     ------------------------------------------------------------

BRA1                ATAGGTATGTTTATGCTATGTTTAATGGGAGCAACAGGAGGAATGCTCTGGTGGTATGAG
BRA1 Sequence 3     ATAGGTATGTTTATGCTATGTTTAATGGGAGCAACAGGAGGAATACTCTGGTGGTATGAG
BRA2 sequence 4.1   ------------------------------------------------------------
BRA1 Sequence 1     ------------------------------------------------------------
BRA2 sequence 5.1   ------------------------------------------------------------
BRA1 Sequence 2     ------------------------------------------------------------
BRA2 sequence 2.1   ------------------------------------------------------------
BRA2 sequence 2.2   ------------------------------------------------------------
BRA2 sequence 4.2   ------------------------------------------------------------
BRA2 sequence 5.2   ------------------------------------------------------------
BRA2 sequence 3.2   ------------------------------------------------------------
BRA2 sequence 6.2   ------------------------------------------------------------
BRA1 Sequence 1     ------------------------------------------------------------
BRA2 sequence 1.1   ------------------------------------------------------------
BRA1 Sequence 4     ------------------------------------------------------------
BRA2 sequence 3.1   ------------------------------------------------------------
BRA1 Sequence 2     ------------------------------------------------------------

BRA1                GATGTGGCACACCCACAATATATAGGATTGATTACTGTTGGTGGGAAATTGGAAGGATCT
BRA1 Sequence 3     GATGTGGCACACCCACAATATATAGGATTGATTACTGTTGGTGGGAAATTGGAAGGATCT
BRA2 sequence 4.1   ------------------------------------------------------------
BRA1 Sequence 1     ------------------------------------------------------------
BRA2 sequence 5.1   ------------------------------------------------------------
BRA1 Sequence 2     ------------------------------------------------------------
BRA2 sequence 2.1   ------------------------------------------------------------
BRA2 sequence 2.2   ------------------------------------------------------------
BRA2 sequence 4.2   ------------------------------------------------------------
BRA2 sequence 5.2   ------------------------------------------------------------
BRA2 sequence 3.2   ------------------------------------------------------------
BRA2 sequence 6.2   ------------------------------------------------------------
BRA1 Sequence 1     ------------------------------------------------------------
BRA2 sequence 1.1   ------------------------------------------------------------
BRA1 Sequence 4     ------------------------------------------------------------
BRA2 sequence 3.1   ------------------------------------------------------------
BRA1 Sequence 2     ------------------------------------------------------------

BRA1                GGAATGACTAGTGCTATAGAATGTTGGGGT-AAGTTTCCTGGCTGTAGACCGTTTGATAA
BRA1 Sequence 3     GGAATGACTAGTGCTATAGAATGTTGGGGTNAAGTTTCCTGGCTGTAGACCGTTTGATAA
BRA2 sequence 4.1   ------------------------------------------------------------
BRA1 Sequence 1     ------------------------------------------------------------
BRA2 sequence 5.1   ------------------------------------------------------------
BRA1 Sequence 2     ------------------------------------------------------------
BRA2 sequence 2.1   ------------------------------------------------------------
BRA2 sequence 2.2   ------------------------------------------------------------
BRA2 sequence 4.2   ------------------------------------------------------------
BRA2 sequence 5.2   ------------------------------------------------------------
BRA2 sequence 3.2   ------------------------------------------------------------
BRA2 sequence 6.2   ------------------------------------------------------------
BRA1 Sequence 1     ------------------------------------------------------------
BRA2 sequence 1.1   ------------------------------------------------------------
BRA1 Sequence 4     ------------------------------------------------------------
BRA2 sequence 3.1   ------------------------------------------------------------
BRA1 Sequence 2     ------------------------------------------------------------

BRA1                TTATTTTAGTTACATGACTAACAGACGTATACATGTACAAAATAACACTGTTTCA-TTAT
BRA1 Sequence 3     TTATTTTAGTTACATGACTAACAGACGTATACATGTACAAAATAACACTGTTTCMWTTAT
BRA2 sequence 4.1   ------------------------------------------------------------
BRA1 Sequence 1     TATATTAGCTTCAGTAACCAGCAGAAGTAGACATGTACAAAATAACACTGTTTCA-TTAT
BRA2 sequence 5.1   ------------------------------------------------------------
BRA1 Sequence 2     ---------------------CAGTAGTAGACATGTACAAAATAACACTGTTTCA-TTAT
BRA2 sequence 2.1   ------------------------------------------------------------
BRA2 sequence 2.2   ------------------------------------------------------------
BRA2 sequence 4.2   ------------------------------------------------------------
BRA2 sequence 5.2   ------------------------------------------------------------
BRA2 sequence 3.2   ------------------------------------------------------------
BRA2 sequence 6.2   ------------------------------------------------------------
BRA1 Sequence 1     ------------------------------------------------------------
BRA2 sequence 1.1   ------------------------------------------------------------
BRA1 Sequence 4     ------------------------------------------------------------
```

```
BRA2 sequence 3.1     ------------------------------------------------------------
BRA1 Sequence 2       ------------------------------------------------------------

BRA1                  TAG-AGTCATATCATAGAGAGATAACATATATATATAAAA-CAATGTGTACAGATAGTGA
BRA1 Sequence 3       TAG-AGTCATATCATAGAGAGATAACATATATATATAAAAACAATGTGTACAGATAGTGA
BRA2 sequence 4.1     ------------------------------------------------------------
BRA1 Sequence 1       TAG-AGTCATATCGTAGAGAGATAGCATTTATATATACAA-CAATGTCTACTGGTAGTGA
BRA2 sequence 5.1     ------------------------------------------------------------
BRA1 Sequence 2       CAGGAGTCATATCATAGAGAGATAACATATATATATACAA-CAATGTGTACTGATAGTGA
BRA2 sequence 2.1     ------------------------------------------------------------
BRA2 sequence 2.2     --------------------------------------------TGTGTACAGATAGTGA
BRA2 sequence 4.2     --------------------------------------------TGTGTACAGATAGTGA
BRA2 sequence 5.2     --------------------------------------------TGTGTACAGATAGTGA
BRA2 sequence 3.2     --------------------------------------------TGTGTACAGATAGTGA
BRA2 sequence 6.2     ---------------------------------------------GTGTACAGATAGTGA
BRA1 Sequence 1       -------------------------------------------------------AGTGA
BRA2 sequence 1.1     ------------------------------------------------------------
BRA1 Sequence 4       ------------------------------------------------------------
BRA2 sequence 3.1     ------------------------------------------------------------
BRA1 Sequence 2       ------------------------------------------------------------

BRA1                  TCATTGTCAAGAA-TAT-GAGTGT---------------AGAAACG--TACAACTGACT-
BRA1 Sequence 3       TCATTGTCAAGAANTATNGAGTGT---------------YMAAGAGGGTACAACTGACTA
BRA2 sequence 4.1     ------------------------------------------------------------
BRA1 Sequence 1       TCATCGTCAAGAT-TAT-GAGKWT---------------RAARAGG--TACAACTGACT-
BRA2 sequence 5.1     ------------------------------------------------------------
BRA1 Sequence 2       TCATTATCAAGAT-TAT-GAGTGT---------------GAGGAGG--TACAACTGACT-
BRA2 sequence 2.1     ------------------------------------------------------------
BRA2 sequence 2.2     TCATTGTCAAGAC-TAT-GAGTGCGAAAGGGTCCAACTAAAAAACA-CTAGTAAATATA-
BRA2 sequence 4.2     TCATTGTCAAGAC-TAT-GAGTGC---------------GAAAGGG--TCCAACTAAAT-
BRA2 sequence 5.2     TCATTGTCAAGAC-TAT-GAGTGC---------------AAAAGGG--TCCAACTAAAA-
BRA2 sequence 3.2     TCATTGTCAASAC-TAT-GAGTGC---------------GAAAGGG--TCCGATTAAAT-
BRA2 sequence 6.2     TCATTGTCAAGAC-TAT-GAGTGC---------------AAAAGGG--TCCAACTAAAA-
BRA1 Sequence 1       TCATTGTCAAGAA-TAT-GAGTGT---------------AAAAAGG--AACAACTGCCT-
BRA2 sequence 1.1     ------------------------------------------------------------
BRA1 Sequence 4       ------------------------------------------------------------
BRA2 sequence 3.1     ------------------------------------------------------------
BRA1 Sequence 2       ------------------------------------------------------------

BRA1                  G----G---------TAAT----GGGTTAAAA-GTG--AATATAA-GCTACACCTTACTA
BRA1 Sequence 3       G----G---------TATR----TGGGTCNAA-GTGTGACTATAAAGCTACAACG--CTA
BRA2 sequence 4.1     ------------------------------------------------------------
BRA1 Sequence 1       A----G---------TART----GGGTCAAGT-GTG--ACTATAA-GCTACAAC--RCTA
BRA2 sequence 5.1     ------------------------------------------------------------
BRA1 Sequence 2       G----G---------TGAG----TAGTTGTAGTGTG--AATATAA-CCTACAAC--ACTA
BRA2 sequence 2.1     ------------------------------------------------------------
BRA2 sequence 2.2     T----A---------ATAA----ATGTGACGG-TTA--GTAATAA-TAGTGGTAATAGTG
BRA2 sequence 4.2     GAGAA----------TAAT----AGTGGAACT-GTG--GAGATTA-GTGGTAA--TAGCA
BRA2 sequence 5.2     G----AGACTAAAAATAAT----ATAATAAAT-GTG--AGTAATA-GTGGTAA--TAGTA
BRA2 sequence 3.2     G----G---------GAATTTTAGTGATAGAA-TAA--GTGTGGA-GGATAATAATAATA
BRA2 sequence 6.2     G----AGACTAAAAATAAT----ATAATAAAT-GTG--AGTAATA-GTGGTAA--TAGTA
BRA1 Sequence 1       A----G---------TAAT----GGGTCCCGT-GTG--AATATAA-GCTACAACG--CTA
BRA2 sequence 1.1     ------------------------------------------------------------
BRA1 Sequence 4       ------------------------------------------------------------
BRA2 sequence3.1      ------------------------------------------------------------
BRA1 Sequence 2       ------------------------------------------------------------

BRA1                  ACACATC--AT----TGGAA-TTTCAAATGGTT--GGTATGTAATCAAACTGAGAATCAA
BRA1 Sequence 3       GCACATC--TTAATTTGGAANTTTCAAATGGGTTGGGTATGTAATCAACTGRAGAATCAA
BRA2 sequence 4.1     ------------------------------------------------------------
BRA1 Sequence 1       GCACATCTTAT----TGGAA-TTTCAAATGGTT--GGTATGTAATCAAACTGAGAATCAA
BRA2 sequence 5.1     ------------------------------------------------------------
BRA1 Sequence 2       MCACATCTTAT----TGGAA-TTTCAAATGGTT--GGTATGTAATCAAAATGAGAATCAA
BRA2 sequence 2.1     ------------------------------------------------------------
BRA2 sequence 2.2     ACACTT---AT----TGGGA-TTTCACATGGTT--ATTATGTAATCAAACTGAGAATAAA
BRA2 sequence 4.2     GCACTT---AT----TGGAA-TTTCACATGGTT--ATTATGTAATCAAACTGAGAATAAA
BRA2 sequence 5.2     GCACTT---AT----TGGGA-TTTCACATGGTT--ATTATGTAATCAAGCTGAGAATACA
BRA2 sequence 3.2     GCACTT---AT----TGGGA-TTTCACATGGTT--ATTATGTAATCAAACTGAGAATAAA
BRA2 sequence 6.2     GCACTT---AT----TGGGA-TTTCACATGGTT--ATTATGTAATCAAGCTGAGAATACA
BRA1 Sequence 1       GCACATCTTAT----TGGAA-TTTCAAATGGTT--GGTATGTAATCAAACTGAGAATCAA
BRA2 sequence 1.1     ------------------------------------------------------------
BRA1 Sequence 4       ------------------------------------------------------------
BRA2 sequence 3.1     ------------------------------------------------------------
BRA1 Sequence 2       ------------------------------------------------------------
```

```
BRA1                  AAGGCAATTC-TTATTCCA-GAAGAAG-AAATGGTACAG---AA-TGATAATAATACTT-
BRA1 Sequence 3       AAGGCAATTCYTTATTCCAAGAAGAAGNAAATGGTACAG---AAATGATAATAATACYTT
BRA2 sequence 4.1     ------------------------------------------------------------
BRA1 Sequence 1       AAGGCAATTC-TTATTCCA-GAAGAAG-AAATGGTACAG---AA-TGATAATAATACTT-
BRA2 sequence 5.1     ------------------------------------------------------------
BRA1 Sequence 2       AAGGCAATTC-TTATTSCA-GAAGAAG-AAGTGGTACAG---AA-TGATAATAATACTT-
BRA2 sequence 2.1     ------------------------------------------------------------
BRA2 sequence 2.2     AAGACAATTC-TTGTTCCA-GAAGAAG-AAATGATACTG---TA-CAATGGCACCACTT-
BRA2 sequence 4.2     AAGACAATTC-TTGTTCCA-GAAGAAG-AAATGATACAGTACAA-TGACAATCGCACTT-
BRA2 sequence 5.2     AAGACAATTC-TTGTTCCA-GAGGAAG-AAATGCAGGAC---AG-TGACAATGGCACTT-
BRA2 sequence 3.2     AAGACGATTC-TTGTTCCA-GAAGAAG-AAATGATACAGTACAA-TGACAATCGCACTT-
BRA2 sequence 6.2     AAGACAATTC-TTGTTCCA-GAGGAAG-AAATGCAGGAC---AG-TGACAATGGCACTT-
BRA1 Sequence 1       AAGGCAATTC-TTATTCCA-GAAGAAG-AAATGGTACAG---AA-TGATAATAATACTT-
BRA2 sequence 1.1     ------------------------------------------------------------
BRA1 Sequence 4       -------------------------------------------------AATAATACTT-
BRA2 sequence 3.1     ------------------------------------------------------------
BRA1 Sequence 2       -------------------------------------------------AATAATACTT-

BRA1                  -GGTCCCCTAGGGGAT-GTAATAGCA--CATGGG-CAAGGGTGAAACATT-GTCC-AGCG
BRA1 Sequence 3       GGGTCCCCTAGGGGATTGTAATAGCCACCATGGGGCAAAGGGGAAACACTTGTCSAGCCA
BRA2 sequence4.1      ------------------------------------------------------------
BRA1 Sequence 1       -GGTCCCCTAGGGGAT-GTAATAGCA--CATGGG-CAAAGGTGAAACATT-GTCC-AGCG
BRA2 sequence5.1      ------------------------------------------------------------
BRA1 Sequence 2       -GGTCTCGTAGGGGAT-GTARTAGCA--CATGGR-CAAAGGTGAAACATT-GTCC-AGCT
BRA2 sequence2.1      ------------------------------------------------------------
BRA2 sequence2.2      -GGGCCCCTAAGGGAT-GTAATGACA--CATGGG-CAAAAGTAAAACATT-GTCC-AATG
BRA2 sequence4.2      -GGGCCCCTAAGGGAT-GTAATGACA--CATGGG-CAAAAGTAAAACATT-GTCC-AATG
BRA2 sequence5.2      -GGGCCCCTAAGGGAT-GTAATGACA--CATGGG-CAAAAGTAAAACATT-GTCC-AATG
BRA2 sequence3.2      -GGGCCCCTAAGGGAT-GTAATGACA--CATGGG-CAAAAGTAAAACATT-GTCC-AATG
BRA2 sequence6.2      -GGGCCCCTAAGGGAT-GTAATGACA--CATGGG-CAAAAGTAAAACATT-GTCC-AATG
BRA1 Sequence 1       NGGTCCCCTAGGGGAT-GTAATAGCA--CATGGG-CAAAGGTGAAACATT-GTCC-AGCG
BRA2 sequence1.1      ------------------------------------------------------------
BRA1 Sequence 4       -GGTCCCCTAGGGGAT-GTAATAGCA--CATGGG-CAAAGGTGAAACATTTGTCCCAGCG
BRA2 sequence3.1      ------------------------------------------------------------
BRA1 Sequence 2       -GGTCCCCTAGGGGAT-GTAATAGCA--CATGGG-CAAAGGTGAAACATTTGTCCCAGCG

BRA1                  GAT-CTGTTGTATGGGATACATCCTATTAGGCTATGTGTGCAGCCACCATTTTTCCTGGT
BRA1 Sequence 3       GATCGGRWTCATGCGATACATCCTATAAGGCCTATGTTGTGCAGCCCACATTTTTCTGGT
BRA2 sequence 4.1     ------------------------------------------------------------
BRA1 Sequence 1       GAT-YTGTTGTATGGGATACATCCTATTAGGCTATGTGTGCAGCCACCATTTTTCCTGGT
BRA2 sequence 5.1     ------------------------------------------------------------
BRA1 Sequence 2       GAT-STGTTKTATGGGATTCATCYTATTAGGCTATGTGTGCAGCCACCATTTTTCTTGGT
BRA2 sequence 2.1     ------------------------------------------------------------
BRA2 sequence 2.2     GAC-TTGTTGTATGGGCTGCATCCTATTAGGTTATGTGTACAGCCACCATTTTTTCTGGT
BRA2 sequence 4.2     GAC-TTGTTGTATGGGCTGCATCCTATTAGGTTATGTGTACAGCCACCATTTTTTCTGGT
BRA2 sequence 5.2     GAC-TTGTTGTATGGGCTGCATCCTATTAGGTTATGTGTACAGCCACCATTTTTTCTGGT
BRA2 sequence 3.2     GAC-TTGTTGTATGGGCTGCATCCTATTAGGTTATGTGTACAGCCACCATTTTTTCTGGT
BRA2 sequence 6.2     GAC-TTGTTGTATGGGCTGCATCCTATTAGGTTATGTGTACAGCCACCATTTTTTCTGGT
BRA1 Sequence 1       GATTYTGTTGTATGGGATACATCCTATTAGGCTATGTGTGCAGCCACCATTTTTCCTGGT
BRA2 sequence 1.1     ------------------------------------------------------------
BRA1 Sequence 4       GAT-TTGTTGTATGGGATACATCCTATTAGGCTATGTGTGCAGCCACCATTTTTCCTGGT
BRA2 sequence 3.1     ------------------------------------------------------------
BRA1 Sequence 2       GAT-TTGTTGTATGGGATACATCCTATTAGGCTATGTGTGCAGCCACCATTTTTCCTGGT

BRA1                  AAAAAATAATGATGGGAACTTCACAAATGGGAACCACACACTTAGCAACTGTGGTCCACA
BRA1 Sequence 3       AAAATATGGATTGGAACTCACGAATATGGGGAACTCAACCACTCCTA-------------
BRA2 sequence 4.1     ------------------------------------------------------------
BRA1 Sequence 1       RAAAAATAATGATGGGAACTTCACAATTGGGAACCACACACTTAGCAACTGTGGTCCACA
BRA2 sequence 5.1     ------------------------------------------------------------
BRA1 Sequence 2       AAAAAATAATGATGGGAACTTCACATTTGGGAACCACACACTYAGCAAYTGTGGTCCACA
BRA2 sequence 2.1     ------------------------------------------------------------
BRA2 sequence 2.2     AAGAAAAGAAGAT------------AATCATAGCCGCACACTTAGTAATTGTGGTCCACA
BRA2 sequence 4.2     AAGAAAAGAAGAT------------AATCATAGCCACACACTTAGTAATTGTGGTCCACA
BRA2 sequence 5.2     AAGAAAAGAAGAT------------AATCATAGCCGCACACTTAGTAATTGTGGTCCACA
BRA2 sequence 3.2     AAGAAAAGAAGAT------------AATCATAGCCGCACACTTAGTAATTGTGGTCCACA
BRA2 sequence 6.2     AAGAAAAGAAGAT------------AATCATAGCCGCACACTTAGTAATTGTGGTCCACA
BRA1 Sequence 1       GAAAAATAATGATGGGAACTTCACAATTGGGAACCACACACTTAGCAACTGTGGTCCACA
BRA2 sequence 1.1     ------------------------------------------------------------
BRA1 Sequence 4       GAAAAATAATGATGGGAACTTCACAATTGGGAACCACACACTTAGCAACTGTGGTCCACA
BRA2 sequence 3.1     ------------------------------------------------------------
BRA1 Sequence 2       GAAAAATAATGATGGGAACTTCACAATTGGGAACCACACACTTAGCAACTGTGGTCCACA

BRA1                  AATATCACTGGGGATATTAGATGAAAATAAAGCTGTTACAAGGGAGGTCAACTGCACTGT
BRA1 Sequence 3       ------------------------------------------------------------
BRA2 sequence 4.1     ------------------------------------------------------------
```

```
BRA1 Sequence 1     AATATCACTGGGGATATTAGATGAAAATAAAGCTGTTACAAGGGGGGTCAACTGCACTGT
BRA2 sequence 5.1   ------------------------------------------------------------
BRA1 Sequence 2     AAYAYMACTGGGGATATTRGATGAAAATAAAGCTGTTACAAGGGGGGTCAACTTCACTGT
BRA2 sequence 2.1   ------------------------------------------------------------
BRA2 sequence 2.2   AATATCATTAGGGATATTAGATAACAATAAAGCTGCTGTAAGAGATGCCAGCTGCCAGGT
BRA2 sequence 4.2   AATATCATTAGGGATATTAGATGACAATAAAGCTGCTGTAAGAGATGCCAACTGCGTGGT
BRA2 sequence 5.2   AATATCACTAGGGATATTAGATAACAATAAAGCTGCTGTAAGAAATGCCAACTGCAGCGT
BRA2 sequence 3.2   AATATCATTAGGGATATTAGATAGCAATAAAGCTGCTGTAAAAGATGCCAGCTGCCAGGT
BRA2 sequence 6.2   AATATCACTAGGGATATTAGATAACAATAAAGCTGCTGTAAGAAATGCCAACTGCAGCGT
BRA1 Sequence 1     AATATCACTGGGGATATTAGATGAAAATAAAGCTGTTACAAGGGGGGTCAACTGCACTGT
BRA2 sequence 1.1   ------------------------------------------------------------
BRA1 Sequence 4     AATATCACTGGGGATATTAGATGAAAATAAAGCTGTTATAAGGGAGGTCAACTGCACTGT
BRA2 sequence 3.1   ------------------------------------------------------------
BRA1 Sequence 2     AATATCACTGGGGATATTAGATGAAAATAAAGCTGTTATAAGGGAGGTCAACTGCACTGT

BRA1                GGCTAAAAAA-GTTTTTCATTTT---AAT---GACTATTCTGGACAGTTTATAAGTCCTA
BRA1 Sequence 3     ------------------------------------------------------------
BRA2 sequence 4.1   ------------------------------------------------------------
BRA1 Sequence 1     GGCTAAAAAA-AGTTTTAATTTT---CAT---GACTATTCTGGACAGTTTATAAGYCCTA
BRA2 sequence 5.1   ------------------------------------------------------------
BRA1 Sequence 2     GGCTAAAAAA-AGTTATAATTTT---CAT---GACTATTCTGGACAGWTTATAAGTCTAA
BRA2 sequence 2.1   ------------------------------------------------------------
BRA2 sequence 2.2   GGAGAGAAAG-ACTGTTCAGATT---CCTAAAGAATATTCTGGACAGCTTATAGTTCCTA
BRA2 sequence 4.2   GGAAAAAAAG-ACTGTTCAGATT---CCTAAAGAATATTCTGGACAGCTTATAGTTCCTA
BRA2 sequence 5.2   AGAGAAAAAG-ACTGTTCAGACT---AAA---GACTATTCTGGACAGCTTATAGTTCCTA
BRA2 sequence 3.2   GGAGAAAAAG-ACTTTTCAGTTTTCTAAA---GGCCATTCTGGACAGCTTATAGTTCCTA
BRA2 sequence 6.2   AGAGAAAAAG-ACTGTTCAGACT---AAA---GACTATTCTGGACAGCTTATAGTTCCTA
BRA1 Sequence 1     GGCTAAAAAAAGTTTTNAATTTT---CAT---GACTATTCTGGACAGTTTATAAGCCCTA
BRA2 sequence 1.1   ------------------------------------------------------------
BRA1 Sequence 4     GGCTAAAAAA-AGTTTTAATTTT---CAT---GACTATTCTGGACAGTTTATAAGTCCTA
BRA2 sequence 3.1   ------------------------------------------------------------
BRA1 Sequence 2     GGCTAAAAAA-AGTTTTAATTTT---CAT---GACTATTCTGGACAGTTTATAAGTCCTA

BRA1                TATTTTATCAATGTAATCTTAGC---CTAACACCTTGTAATGGGACAAACTCACC-----
BRA1 Sequence 3     ------------------------------------------------------------
BRA2 sequence 4.1   ------------------------------------------------------------
BRA1 sequence 1     TATTTTATCAATGTAATCTTAGC---CTAAAACCTTGTAATGGGACAAACTCATC-----
BRA2 sequence 5.1   ------------------------------------------------------------
BRA1 Sequence 2     TATTTTATCAATGTAATCTTAGY---CTAAAMCCTTGTAATGGGACAAACTCATC-----
BRA2 sequence 2.1   ------------------------------------------------------------
BRA2 sequence 2.2   TATTCTATAAGTGTACTATTA-----CTAATCTGACAAGGTGCTATAACAACAGCTCAAC
BRA2 sequence 4.2   TATTCTATAAGTGTACTATTA-----CTAATCTGACAAGGTGCAATAACGACAACTCAAC
BRA2 sequence 5.2   TATTCTATAAGTGTACTATTACTGGTCTGACAAGGTGCGATAACAACAACTCAAC-----
BRA2 sequence 3.2   TATTCTATAAGTGTACTATTACTAATCTGACAAGGTGCTATAACAACAGCTCAAC-----
BRA2 sequence 6.2   TATTCTATAAGTGTACTATTACTGGTCTGACAAGGTGCGATAACAACAACTCAAC-----
BRA1 Sequence 1     TATTTTATCAATGTAATCTTAGC---CTAAAACCTTGTAATGGGACAAACTCATC-----
BRA2 sequence 1.1   ------------------------------------------------------------
BRA1 Sequence 4     TATTTTATCAATGTAATCTTAGC---CTAAAACCTTGTAATGGGACAAACTCATC-----
BRA2 sequence 3.1   ------------------------------------------------------------
BRA1 Sequence 2     TATTTTATCAATGTAATCTTAGC---CTAAAACCTTGTAATGGGACAAACTCATC-----

BRA1                AGTAAGTATTATCAGATATGATGAGGACACTGTTGAATATTTGATAAGAAACACTGTTGA
BRA1 Sequence 3     ------------------------------------------------------------
BRA2 sequence 4.1   ------------------------------------------------------------
BRA1 Sequence 1     AGTAAGTATTATCAGATATGATGAG------------------------AACACTGTTGA
BRA2 sequence 5.1   ------------------------------------------------------------
BRA1 Sequence 2     AGTAAGTATTATCAGATATGATGAGGACGCTGTYGAATATGTGATA--------------
BRA2 sequence 2.1   ------------------------------------------------------------
BRA2 sequence 2.2   AATAAGTATTATCAGGTATGATGAGAAAAATGTTCAATATTTGTTATGTAAAACAAATAA
BRA2 sequence 4.2   AATAAGTATTATCAGATATGATAAGGACAATGTTCAATATTTGTTATGTAAAAATAATAC
BRA2 sequence 5.2   AATAAGTGTTATCAGGTATGATGAGAACAGTGTTCAATATTTGTTATGTAAAACAAATAA
BRA2 sequence 3.2   AATAAGTATTATCAGGTATGATGAG-----------------------AAAAA-TGTTCA
BRA2 sequence 6.2   AATAAGTGTTATCAGGTATGATGAGAACAGTGTTCAATATTTGTTATGTAAAACAAATAA
BRA1 Sequence 1     AGTAAGTATTATCAGATATGATGAG-----------------------AA-CACTGTTGA
BRA2 sequence 1.1   ------------------------------------------------------------
BRA1 Sequence 4     AGTAAGTATTATCAGATATGATGAGAACACTGTTGAGTATTTGATA--------------
BRA2 sequence 3.1   ------------------------------------------------------------
BRA1 Sequence 2     AGTAAGTATTATCAGATATGATGAGAACACTGTTGAGTATTTGATA--------------

BRA1                ATATTTGATATGCAAGGCTAAAAACATCACTGAAAA------------A-----------
BRA1 Sequence 3     ------------------------------------------------------------
BRA2 sequence 4.1   ------------------------------------------------------------
BRA1 Sequence 1     ATATTTGATATGCAAGGCTAATAGCACCACTGAAAACACCACTGAAGGA-----------
BRA2 sequence 5.1   ------------------------------------------------------------
BRA1 Sequence 2     ----------TGCAAGGMTAATAGCAACACTGAAAACACCACTGAAGGA-----------
```

```
BRA2 sequence 2.1   ------------------------------------------------------------
BRA2 sequence 2.2   TACAGATGCAGGTAAT--------------------------------------------
BRA2 sequence 4.2   AGATGCAGGT--------------------------------------------------
BRA2 sequence 5.2   TACAGATGCAGGTAAT--------------------------------------------
BRA2 sequence 3.2   ATATTTGTTATGTAAAACAAATAATACAGATGCAGG------------T-----------
BRA2 sequence 6.2   TACAGATGCAGGTAAT--------------------------------------------
BRA1 Sequence 1     ATATTTGATATGCAAGGCTAATAGCACCACTGAAAACACCACTGAAGGA-----------
BRA2 sequence 1.1   ------------------------------------------------------------
BRA1 Sequence 4     ----------TGCAAGGCTAATAACATCACT-AAAA------------ACACCGCTGAAA
BRA2 sequence 3.1   ------------------------------------------------------------
BRA1 Sequence 2     ----------TGCAAGGCTAATAACATCACT-AAAA------------ACACCGCTGAAA

BRA1                --------------AACTTTTCATGTGTAGTACAAACTTTTGGAACAATAGGACAGGCAC
BRA1 Sequence 3     ------------------------------------------------------------
BRA2 sequence 4.1   ------------------------------------------------------------
BRA1 Sequence 1     --------------AGCTTTTCATGTGTAGTACAAACTTTTGGAACAATAGGACAGGCAC
BRA2 sequence 5.1   ------------------------------------------------------------
BRA1 Sequence 2     --------------AACTTTTCATGTGTAGTACAAACTTTTGGAACAATAGGACAGGCAC
BRA2 sequence 2.1   ------------------------------------------------------------
BRA2 sequence 2.2   -----------------TTTTCATGTGTAGTACAAACTTTTGGGAAAATAGGACAGGCAC
BRA2 sequence 4.2   --------------AATTTTTCATGTGTAGTACAAACTTTTGGGAAAATAGGACAGGCAC
BRA2 sequence 5.2   -----------------TTTTCATGTGTAGTACAAACTTTTGGGAAAATAGGACAGGCAC
BRA2 sequence 3.2   --------------AATTTTTCATGTGTAGTACAAACTTTTGGAAAAATAGGACAGGCAC
BRA2 sequence 6.2   -----------------TTTTCATGTGTAGTACAAACTTTTGGGAAAATAGGACAGGCAC
BRA1 Sequence 1     --------------AGCTTTTCATGTGTAGTACAAACTTTTGGAACAATAGGACAGGCAC
BRA2 sequence 1.1   ------------------------------------------------------------
BRA1 Sequence 4     GCACCACTGAAGGAAACTTTTCATGTGTAGTACAAACTTTTGGAACAATAGGACAGGCAC
BRA2 sequence 3.1   ------------------------------------------------------------
BRA1 Sequence 2     GCACCACTGAAGGAAACTTTTCATGTGTAGTACAAACTTTTGGAACAATAGGACAGGCAC

BRA1                ATATAGAACTACCCAGAAAAAATAAAAAGATAATTGGAAACCAGTTCACACAATATAATT
BRA1 Sequence 3     ------------------------------------------------------------
BRA2 sequence 4.1   ------------------------------------------------------------
BRA1 Sequence 1     ATATAGAACTACCCAGAAAAAATAAAAAGATAATTGGAGACCGGTTCACACAATATAATT
BRA2 sequence 5.1   ------------------------------------------------------------
BRA1 Sequence 2     ATATAGAACTACCCAGAAAAAATAAAAAGATAATTGGAAACCAGTTCACACAATATGATT
BRA2 sequence 2.1   ------------------------------------------------------------
BRA2 sequence 2.2   ATATAGAGTTACCCAGAAACAATAAAAGGATAAGAGAGAACAAATTCACACAGTATAATT
BRA2 sequence 4.2   ATATAGAGTTACCCAGAAACAATAAAAGGATAAGAGAGAACAAATTCACACAGTATAATT
BRA2 sequence 5.2   ATATAGAGTTACCCAGAAACAATAAAAGGATAAGAGAGAACAAATTCACACAGTATAATT
BRA2 sequence 3.2   ATATAGAGTTACCCAGAAACAATAAAAGGATAAGAGAGAACAAATTCACACAGTATAATT
BRA2 sequence 6.2   ATATAGAGTTACCCAGAAACAATAAAAGGATAAGAGAGAACAAATTCACACAGTATAATT
BRA1 Sequence 1     ATATAGAACTACCCAGAAAAAATAAAAAGATAATTGGAGACCGGTTCACACAATATAATT
BRA2 sequence 1.1   ------------------------------------------------------------
BRA1 Sequence 4     ATATAGAACTACCCAGAAAAAATAAAAAGATAATTGGAGACCGGTTCACACAATATAATT
BRA2 sequence 3.1   ------------------------------------------------------------
BRA1 Sequence 2     ATATAGAACTACCCAGAAAAAATAAAAAGATAATTGGAGACCGGTTCACACAATATAATT

BRA1                GTTCAATAAATGATAAGACAGAGTTAGAAAAATGGAAGTTGGTAAAAA-GTTCAGGTATT
BRA1 Sequence 3     ------------------------------------------------------------
BRA2 sequence 4.1   ------------------------------------------------------------
BRA1 Sequence 1     GTTCAATAAATAATAAGACAGAGTTAGAAAAATGGAAGTTGGTAAAAA-GTTCAGGTATT
BRA2 sequence 5.1   ------------------------------------------------------------
BRA1 Sequence 2     GTTCAATAAATAATAAGACAGAGTTAGAAAAATGGAAGTN--------------------
BRA2 sequence 2.1   ------------------------------------------------------------
BRA2 sequence 2.2   GTTCAATAAATAATCAGACAGAGTTAAAAGAATGGAAATTGATAAGAG-GTTCCGGTATT
BRA2 sequence 4.2   GTTCAATAAATAATCAGACAGAGTTAAAAGAATGGAAATTGATAAAAG-GTTCCGGTATT
BRA2 sequence 5.2   GTTCAATAAATAATCAGACAGAGTTAAAAGAATGGAAATTGATAAAAG-GTTCCGGTATT
BRA2 sequence 3.2   GTTCAATAAATAATCAGACAGAGTTAAAAGAATGGAAATTGATAAAAG-GTTCCGGTATT
BRA2 sequence 6.2   GTTCAATAAATAATCAGACAGAGTTAAAAGAATGGAAATTGATAAAAG-GTTCCGGTATT
BRA1 Sequence 1     GTTCAATAAATAATAAGACAGAGTTAGAAAAATGGAAGTTGGTAAAAANGTTCAGGTATT
BRA2 sequence 1.1   ------------------------------------------------------------
BRA1 Sequence 4     GTTCAATAAATGATAAGACAGAGTTAGAAAAATGGAAGTTGGTAAAAA-GTTCAGGTATT
BRA2 sequence 3.1   ------------------------------------------------------------
BRA1 Sequence 2     GTTCAATAAATGATAAGACAGAGTTAGAAAAATGGAAGTTGGTAAAAA-GTTCAGGTATT

BRA1                ACTCCTTTTCCTGTTACTTCTCGGGCCAATACAGGACTAGTTAGATACAAAAGAGATTTT
BRA1 sequence 3     ------------------------------------------------------------
BRA2 sequence 4.1   ------------------------------------------------------------
BRA1 Sequence 1     ACTCCTTTTCCTGTTACTTCTCGGGC----------------------------------
BRA2 sequence 5.1   ------------------------------------------------------------
BRA1 Sequence 2     ------------------------------------------------------------
BRA2 sequence 2.1   ------------------------------------------------------------
BRA2 sequence 2.2   ACTCCTATTCCTATTACTTCTCAGGCTAACACAGGATTAATTAGATACAAAAGAGATTTT
BRA2 sequence 4.2   ACTCCTGTTCCTATTACTTCTCAGGCTAACACAGGATTAATTAGATACAAAAGAGATTTT
```

```
BRA2 sequence 5.2   ACTCCTATTCCTATTACTTCTCAGGCTAACACAGGATTAATTAGATACAAAAGAGATTTT
BRA2 sequence 3.2   ACTCCTATTCCTATTACTTCTCAGGCTAACACAGGATTAATTAGATACAAAAGAGATTTT
BRA2 sequence 6.2   ACTCCTATTCCTATTACTTCTCAGGCTAACACAGGATTAATTAGATACAAAAGAGATTTT
BRA1 Sequence 1     ACTCCTTTTCCTGTTACTTCTCGGGC----------------------------------
BRA2 sequence 1.1   ------------------------------------------------------------
BRA1 Sequence 4     ACTCCTTTTCCTGTTACTTCTCGGGC----------------------------------
BRA2 sequence 3.1   ------------------------------------------------------------
BRA1 Sequence 2     ACTCCTTTTCCTGTTACTTCTCGGGC----------------------------------

BRA1                GGAATATCAGCAATAGTGGCGGCAATAGTGGCTGCAACTGCTATTGCTGCTAGTGCTACT
BRA1 Sequence 3     ------------------------------------------------------------
BRA2 sequence 4.1   ---------------------------------------GCTATTGCTGCTAGTGCTACC
BRA1 Sequence 1     ------------------------------------------------------------
BRA2 sequence 5.1   ------------------------------------------------------------
BRA1 Sequence 2     ------------------------------------------------------------
BRA2 sequence 2.1   ------------------------------------------------------------
BRA2 sequence 2.2   GGAATATCAGCAATAGTGGCGGCAATAGTGGCTGCAACCGCTATTGCTGCTAGTGCTACC
BRA2 sequence 4.2   GGAATATCAGCAATAGTGGCGGCAATAGTGGCTGCAACCGCTATTGCTGCTAGTGCTACC
BRA2 sequence 5.2   GGAATATCAGCAATAGTGGCGGCAATAGTGGCTGCAACCGCTATTGCTGCTAGTGCTACC
BRA2 sequence 3.2   GGAATATCAGCAATAGTGGCGGCAATAGTGGCTGCAACCGCTATTGCTGCTAGTGCTACC
BRA2 sequence 6.2   GGAATATCAGCAATAGTGGCGGCAATAGTGGCTGCAACCGCTATTGCTGCTAGTGCTACC
BRA1 Sequence 1     ------------------------------------------------------------
BRA2 sequence 1.1   ------------------------------------------------------------
BRA1 Sequence 4     ------------------------------------------------------------
BRA2 sequence 3.1   ------------------------------------------------------------
BRA1 Sequence 2     ------------------------------------------------------------

BRA1                ATGTCTTATATTGCCTTAACTGAAACAAATAAAATGAGTGATGTAATGAATCATACATTT
BRA1 Sequence 3     ------------------------------------------------------------
BRA2 sequence 4.1   ATGTCTTATATTGCCTTGACTGAAGCAAGTAAAGTTACTAATGCCATGAATCATACATTT
BRA1 Sequence 1     ------------------------------------------------------------
BRA2 sequence 5.1   --------------------------------------TAATGCCATGAATCATACMTTT
BRA1 Sequence 2     ------------------------------------------------------------
BRA2 sequence 2.1   ---------------------------------------------------------TTT
BRA2 sequence 2.2   ATGTCTTATATTGCTTTGACTGAAGCAAGTAAAGTTACTAATGCCATGAATCATACCTTT
BRA2 sequence 4.2   ATGTCTTATATTGCTTTGACTGAAGCAAGTAAAGTTACTAATGCCATGAATCATACCTTT
BRA2 sequence 5.2   ATGTCTTATATTGCTTTGACTGAGGCAAGTAAAGTTAGTAATGCCATGAATCATACATTT
BRA2 sequence 3.2   ATGTCTTATATTGCTTTGACTGAAGCAAGTAAAGTTAGTAATGCCATGAATCATACATTT
BRA2 sequence 6.2   ATGTCTTATATTGCTTTGACTGAGGCAAGTAAAGTTAGTAATGCCATGAATCATACATTT
BRA1 Sequence 1     ------------------------------------------------------------
BRA2 sequence 1.1   ------------------------------------------------------------
BRA1 Sequence 4     ------------------------------------------------------------
BRA2 sequence 3.1   ------------------------------------------------------------
BRA1 Sequence 2     ------------------------------------------------------------

BRA1                GTAGTGGAAAATGATACAATCAGAGCAACGGAAATGATAGAAAAACAGATACATATATTA
BRA1 Sequence 3     ------------------------------------------------------------
BRA2 sequence 4.1   GAAGTAGAGAATAACACAATCCATGCAACAGAGATGATAGAAAAACAAATGCATATATTA
BRA1 Sequence 1     ------------------------------------------------------------
BRA2 sequence 5.1   GAAGTAGAGAATAACACAATCCATGCAACAGAGATGATAGAAAAACAAATGCATATATTA
BRA1 Sequence 2     ------------------------------------------------------------
BRA2 sequence 2.1   GAAGTAGAGAATAACACAATCCATGCAACAGAGATGATAGAAAAACAAATGCATATATTA
BRA2 sequence 2.2   GAAGTAGAGAATAACACAATCCATGCAACAGAGATGATAGAAAAACAAATGCATATATTA
BRA2 sequence 4.2   GAAGTAGAGAATAACACAATCCATGCAACAGAGATGATAGAAAAACAAATGCACATATTA
BRA2 sequence 5.2   GAGGTAGAGAATAACACAATCCATGCAACAGAGATGATAGAAAAACAAATGCATATATTA
BRA2 sequence 3.2   GAGGTAGAGAATAACACAATCCATGCAACAGAGATGATAGAAAAACAAATGCATATATTA
BRA2 sequence 6.2   GAGGTAGAGAATAACACAATCCATGCAACAGAGATGATAGAAAAACAAATGCATATATTA
BRA1 Sequence 1     ------------------------------------------------------------
BRA2 sequence 1.1   ---GTAGAGAATAACACAATCCATGCAACAGAGATGATAGAAAAACAAATGCATRTATTA
BRA1 Sequence 4     ------------------------------------------------------------
BRA2 sequence 3.1   ----------------------------------------AAAAACAAATGCATATATTA
BRA1 Sequence 2     ------------------------------------------------------------

BRA1                TATGCAATGGTTCTTCAGACCCATGCAGATGTCC-AGTTACTAAAAGAAAGACAAAAAAT
BRA1 Sequence 3     ------------------------------------------------------------
BRA2 sequence 4.1   TATGCAATGATTCTTCAGACCCATGCAGATGTCC-AGTTACTAAAAGAAAGACAAAAAAT
BRA1 Sequence 1     ------------------------------------------------------------
BRA2 sequence 5.1   TATGCAATGATTCTTCAGACCCATGCAGATGTCC-AGTTACTAAAAGAAAGACAAAAAAT
BRA1 Sequence 2     ------------------------------------------------------------
BRA2 sequence 2.1   TATGCAATGATTCTTCAGACCCATGCAGATGTCC-AGTTACTAAAAGAAAGACAAAAAAT
BRA2 sequence 2.2   TATGCAATGATTCTTCAGACCCATGCAGATGTCC-AGTTACTAAA----------------
BRA2 sequence 4.2   TATGCAATGATTCTTCAGACCCATGCAGATGTCC-AGTTACTAAA----------------
BRA2 sequence 5.2   TATGCAATGATTCTTCAGACCCATGCAGATGTCC-AGTTA---------------------
BRA2 sequence 3.2   TATGCAATGATTCTTCAGACCCATGCAGATGTCC-AGTTACTAAA----------------
BRA2 sequence 6.2   TATGCAATGATTCTTCAGACCCATGCAGATGTCC-AG------------------------
```

```
BRA1 Sequence 1      ------------------------------------------------------------
BRA2 sequence 1.1    TATGCAATGATTCTTCAGACCCATGCAGATGTCC-AGTTACTAAAAGAAAGACAAAAAAT
BRA1 Sequence 4      ------------------------------------------------------------
BRA2 sequence 3.1    TATGCAATGATTCTTCAGACCCATGCAGATGTCCCAGTTACTAAAAGAAAGACAAAAAAT
BRA1 Sequence 2      ------------------------------------------------------------

BRA1                 AGAGGAAACATTTAATTTG-ATAGGGTGTGTTGAGAGGTCACATACATTCTGCCATACTG
BRA1 Sequence 3      ------------------------------------------------------------
BRA2 sequence 4.1    AGAGGAAACATTTAATTTG-ATAGGGTGTGTTGAAAGATCACATACATTCTGCCATACTG
BRA1 Sequence 1      ------------------------------------------------------------
BRA2 sequence 5.1    AGATGAAACATTTAATTTG-ATAGGGTGTGTTGAAAGATCACATACATTCTGTCATACTG
BRA1 Sequence 2      ------------------------------------------------------------
BRA2 sequence 2.1    AGATGAAACATTTAATTTG-ATAGGGTGTGTTGAAAGATCACATACATTCTGTCATACTG
BRA2 sequence 2.2    ------------------------------------------------------------
BRA2 sequence 4.2    ------------------------------------------------------------
BRA2 sequence 5.2    ------------------------------------------------------------
BRA2 sequence 3.2    ------------------------------------------------------------
BRA2 sequence 6.2    ------------------------------------------------------------
BRA1 Sequence 1      ------------------------------------------------------------
BRA2 sequence 1.1    AGATGAAACATTTAATTTG-ATAGGGTGTGTTGAAAGATCACATACATTCTGTCATACTG
BRA1 Sequence 4      ------------------------------------------------------------
BRA2 sequence 3.1    AGATGAAACATTTAATTTG-ATAGGGTGTGTTGAAAGATCACATACATTCTGTCATACTG
BRA1 Sequence 2      ------------------------------------------------------------

BRA1                 GACA-TCCATGGAATGAATCTTGGGGGCAAT-TGAATGAAACAACAGAATGGGA
BRA1 Sequence 3      ------------------------------------------------------
BRA2 sequence 4.1    GACA-TCCATGGAATGATTCTTGGGGACAAT-TAAATGACTCAACACAATGGGA
BRA1 Sequence 1      ------------------------------------------------------
BRA2 sequence 5.1    GACA-TCCATGGAATGATTCTTGGGGACAAT-TAAATGACTCAACACAATGGGA
BRA1 Sequence 2      ------------------------------------------------------
BRA2 sequence 2.1    GACA-TCCATGGAATGATTCTTGGGGACAAT-TAAATGACTCAACACAATGGGA
BRA2 sequence 2.2    ------------------------------------------------------
BRA2 sequence 4.2    ------------------------------------------------------
BRA2 sequence 5.2    ------------------------------------------------------
BRA2 sequence 3.2    ------------------------------------------------------
BRA2 sequence 6.2    ------------------------------------------------------
BRA1 Sequence 1      ------------------------------------------------------
BRA2 sequence 1.1    GACA-TCCATGGAATGATTCTTGGGGACAAT-TAAATGACTCAACACAATGGGA
BRA1 Sequence 4      ------------------------------------------------------
BRA2 sequence 3.1    GACA-TCCATGGAATGATTCTTGGGGACAAT-TAAATGACTCAACACAATGGGA
BRA1 Sequence 2      ------------------------------------------------------

BRA1                 TGATTGGATAACTAAAATGAATAGGCTTGAAACGGAGATATTGACTACTCTGCATGCAGC
BRA1 Sequence 3      ------------------------------------------------------------
BRA2 sequence 4.1    TGATTGGGTAAACAAAATGAATGAGTATGAACAGGACATATTAACAACTCTTCATGCAGC
BRA1 Sequence 1      ------------------------------------------------------------
BRA2 sequence 5.1    TGATTGGGTAAACAAAATGAATGAGTATGAACAGGACATATTAACAACTCTTCATGCAGC
BRA1 Sequence 2      ------------------------------------------------------------
BRA2 sequence 2.1    TGATTGGGTAAACAAAATGAATGAGTATGAACAGGACATATTAACAACTCTTCATGCAGC
BRA2 sequence 2.2    ------------------------------------------------------------
BRA2 sequence 4.2    ------------------------------------------------------------
BRA2 sequence 5.2    ------------------------------------------------------------
BRA2 sequence 3.2    ------------------------------------------------------------
BRA2 sequence 6.2    ------------------------------------------------------------
BRA1 Sequence 1      ------------------------------------------------------------
BRA2 sequence 1.1    TGATTGGGTAAACAAAATGAATGAGTATGAACAGGACATATTAACAACTCTTCATGCAGC
BRA1 Sequence 4      ------------------------------------------------------------
BRA2 sequence 3.1    TGATTGGGTAAACAAAATGAATGAGTATGAACAGGACATATTAACAACTCTTCATGCAGC
BRA1 Sequence 2      ------------------------------------------------------------


BRA1                 TAAAAATAATTTAGAACAAGCGATGATTACTTTTAATACACCTGATAGTATAGCTCAATT
BRA1 Sequence 3      ------------------------------------------------------------
BRA2 sequence 4.1    TAGAAATAATTTAGAACAGGCTATGATTACTTTTAATACACCTGATAGTATAGCTCAATT
BRA1 Sequence 1      ------------------------------------------------------------
BRA2 sequence 5.1    TAGAAATAATTTAGAACAGGCTATGATTACTTTTAATACACCTGATAGTATAGCTCAATT
BRA2 sequence 2.1    TAGAAATAATTTAGAACAGGCTATGATTACTTTTAATACACCTGATAGTATAGCTCAATT
BRA2 sequence 2.2    ------------------------------------------------------------
BRA2 sequence 4.2    ------------------------------------------------------------
BRA2 sequence 5.2    ------------------------------------------------------------
BRA2 sequence 3.2    ------------------------------------------------------------
BRA2 sequence 6.2    ------------------------------------------------------------
BRA1 Sequence 1      ------------------------------------------------------------
BRA2 sequence 1.1    TAGAAATAATTTAGAACAGGCTATGATTACTTTTAATACACCTGATAGTATAGCTCAATT
BRA1 Sequence 4      ------------------------------------------------------------
```

```
BRA2 sequence 3.1   TAGAAATAATTTAGAACAGGCTATGATTACTTTTAATACACCTGATAGTATAGCTCAATT
BRA1 Sequence 2     ------------------------------------------------------------

BRA1                TGGAAGAAATATTTGGAGTCATATAGCAAACTGGATTCCGGGACTAGGAGCTTCTATAAT
BRA1 Sequence 3    ------------------------------------------------------------
BRA2 sequence 4.1   CGGAAAAAAATATTTGGAGTCATATAGCAAACTGGATTCCAGGACTGGGAGCTTCAATAAT
BRA1 Sequence 1    ------------------------------------------------------------
BRA2 sequence 5.1   TGGAAAAAAATATTTGGAGTCATATAGCAAACTGGATTCCAGGACTGGGAGCTTCAATAAT
BRA1 Sequence 2    ------------------------------------------------------------
BRA2 sequence 2.1   TGGAAAAAAATATTTGGAGTCATATAGCAAACTGGATTCCAGGACTGGGAGCTTCAATAAT
BRA2 sequence 2.2   ------------------------------------------------------------
BRA2 sequence 4.2   ------------------------------------------------------------
BRA2 sequence 5.2   ------------------------------------------------------------
BRA2 sequence 3.2   ------------------------------------------------------------
BRA2 sequence 6.2   ------------------------------------------------------------
BRA1 Sequence 1    ------------------------------------------------------------
BRA2 sequence 1.1   YGGAAAAAAATATTTGGAGTCATATAGCAAACTGGATTCCAGGACTGGGAGCTTCAATAAT
BRA1 Sequence 4    ------------------------------------------------------------
BRA2 sequence 3.1   YGGAAAAAAATATTTGGAGTCATATAGCAAACTGGATTCCAGGACTGGGAGCTTCAATAAT
BRA1 Sequence 2    ------------------------------------------------------------

BRA1                AAAATATATAATAATGTTGTTATTGTGTTATGTGGTACTAACCTCTTCACCTAAGATCCT
BRA1 Sequence 3    ------------------------------------------------------------
BRA2 sequence 4.1   AAAATATATAGTAATGCTATTATTGTGTTATGTGGTACTAACCTCTTCACCTAAGATCCC
BRA1 Sequence 1    ------------------------------------------------------------
BRA2 sequence 5.1   AAAATATATAGTAATGCTATTATTGTGTTATGTGGTACTAACCTCTTCACCTAAGATCCT
BRA1 Sequence 2    ------------------------------------------------------------
BRA2 sequence 2.1   AAAATATATAGTAATGCTATTATTGTGTTATGTGGTACTAACCTCTTCACCTAAGATCCT
BRA2 sequence 2.2   ------------------------------------------------------------
BRA2 sequence 4.2   ------------------------------------------------------------
BRA2 sequence 5.2   ------------------------------------------------------------
BRA2 sequence 3.2   ------------------------------------------------------------
BRA2 sequence 6.2   ------------------------------------------------------------
BRA1 Sequence 1    ------------------------------------------------------------
BRA2 sequence 1.1   AAAATATATAGTAATGCTATTATTGTGTTATGTGGTACTAACCTCTTCACCTAAGATCCT
BRA1 Sequence 4    ------------------------------------------------------------
BRA2 sequence 3.1   AAAATATATAGTAATGCTATTATTGTGTTATGTGGTACTAACCTCTTCACCTAAGATCCT
BRA1 Sequence 2    ------------------------------------------------------------

BRA1                CAGAAACCTCTTGAGAATGATGAGTGGTGCAGGATCCTCCGTCAATCGCTACCTGAAGAG
BRA1 Sequence 3    ------------------------------------------------------------
BRA2 sequence 4.1   CAGAAACCTCTTGACAACGATGAGTGGTGCAGGATCCTCCGTCAATCACTTCCTGAAGAA
BRA1 Sequence 1    ------------------------------------------------------------
BRA2 sequence 5.1   CAGAAACCTCTTGACAACGATGAGTGGTGCAGGATCCTCCGTCAATCACTTCCTGAAGAA
BRA1 Sequence 2    ------------------------------------------------------------
BRA2 sequence 2.1   CAGAAACCTCTTGACAACGATGAGTGGTGCAGGATCCTCCGTCAATCACTTCCTGAAGAA
BRA2 sequence 2.2   ------------------------------------------------------------
BRA2 sequence 4.2   ------------------------------------------------------------
BRA2 sequence 5.2   ------------------------------------------------------------
BRA2 sequence 3.2   ------------------------------------------------------------
BRA2 sequence 6.2   ------------------------------------------------------------
BRA1 Sequence 1    ------------------------------------------------------------
BRA2 sequence 1.1   CAGAAACCTCTTGACAACGATGAGTGGTGCAGGATCCTCCGTCAATCACTTCCTGAAGAA
BRA1 Sequence 4    ------------------------------------------------------------
BRA2 sequence 3.1   CAGAAACCTCTTGACAACGATGAGTGGTGCAGGATCCTCCGTCAATCACTTCCTGAAGAA
BRA1 Sequence 2    ------------------------------------------------------------

BRA1                ACAACACCATCAGAAACATGCATGGAAAGAAGATGCCTGGGACCAGGACCAGTACAGAAT
BRA1 Sequence 3    ------------------------------------------------------------
BRA2 sequence 4.1   GAAACACCATCAGAAACATGCATGGAAAGAAGAATCCTGGGACCAGGACCAGTACAGTGT
BRA1 Sequence 1    ------------------------------------------------------------
BRA2 sequence 5.1   GAAACACCATCAGAAACATGCATGGAAAGAAGAATCCTGGGACCAGGACCAGTACAGTGT
BRA1 Sequence 2    ------------------------------------------------------------
BRA2 sequence 2.1   GAAACACCATCAGAAACATGCATGGAAAGAAGAATCCTGGGACCAGGACCAGTACAGTGT
BRA2 sequence 2.2   ------------------------------------------------------------
BRA2 sequence 4.2   ------------------------------------------------------------
BRA2 sequence 5.2   ------------------------------------------------------------
BRA2 sequence 3.2   ------------------------------------------------------------
BRA2 sequence 6.2   ------------------------------------------------------------
BRA1 Sequence 1    ------------------------------------------------------------
BRA2 sequence 1.1   GAAACACCATCAGAAACATGCATGGAAAGAAGAATCCTGGGACCAGGACCAGTACAGTGT
BRA1 Sequence 4    ------------------------------------------------------------
BRA2 sequence 3.1   GAAACACCATCAGAAACATGCATGGAAAGAAGAATCCTGGGACCAGGACCAGTACAGTGT
BRA1 Sequence 2    ------------------------------------------------------------
```

```
BRA1                   ACACCTAGCAGACGCGACCGGTGGCTCAGAGGACAAATACAACAAGCCGAAATGCTCCAA
BRA1 Sequence 3        ------------------------------------------------------------
BRA2 sequence 4.1      ACACCTAGCAGGCGTAACCGGTGGATTAGAGGACAAATACAACAAGCTGAAATACTCCAA
BRA1 Sequence 1        ------------------------------------------------------------
BRA2 sequence 5.1      ACACCTAGCAGGCGTAACCGGTGGATTAGAGGACAAATACAACAAGCTGAAATACTCCAA
BRA1 Sequence 2        ------------------------------------------------------------
BRA2 sequence 2.1      ACACCTAGCAGGCGTAACCGGTGGATTAGAGGACAAATACAACAAGCTGAAATACTCCAA
BRA2 sequence 2.2      ------------------------------------------------------------
BRA2 sequence 4.2      ------------------------------------------------------------
BRA2 sequence 5.2      ------------------------------------------------------------
BRA2 sequence 3.2      ------------------------------------------------------------
BRA2 sequence 6.2      ------------------------------------------------------------
BRA1 Sequence 1        ------------------------------------------------------------
BRA2 sequence 1.1      ACACCTAGCAGGCGTAACCGGTGGATTAGAGGACAAATACAACAAGCTGAAATACTCCAA
BRA1 Sequence 4        ------------------------------------------------------------
BRA2 sequence 3.1      ACACCTAGCAGGCGTAACCGGTGGATTAGAGGACAAATACAACAAGCTGAAATACTCCAA
BRA1 Sequence 2        ------------------------------------------------------------

BRA1                   GAGAAGTTACARTGGCGGATTAAAGGGGTTCAACAGACAGCCAAAGAATTGGAAGAACTC
BRA1 Sequence 3        ------------------------------------------------------------
BRA2 sequence 4.1      GAGAAATTACAATGGCGGATCAAAGGAGTTCAACAAACGGCCCAAGAGCTGGGAGACCTT
BRA1 Sequence 1        ------------------------------------------------------------
BRA2 sequence 5.1      GAGAAATTACAATGGCGGATCAAAGGAGTTCAACAAACGGCCCAAGAGCTGGGAGACCTT
BRA1 Sequence 2        ------------------------------------------------------------
BRA2 sequence 2.1      GAGAAATTACAATGGCGGATCAAAGGAGTTCAACAAACGGCCCAAGAGCTGGGAGACCTT
BRA2 sequence 2.2      ------------------------------------------------------------
BRA2 sequence 4.2      ------------------------------------------------------------
BRA2 sequence 5.2      ------------------------------------------------------------
BRA2 sequence 3.2      ------------------------------------------------------------
BRA2 sequence 6.2      ------------------------------------------------------------
BRA1 Sequence 1        ------------------------------------------------------------
BRA2 sequence 1.1      GAGAAATTACAATGGCGGATCAAAGGAGTTCAACAAACGGCCCAAGAGCTGGGAGACCTT
BRA1 Sequence 4        ------------------------------------------------------------
BRA2 sequence 3.1      GAGAAATTACAATGGCGGATCAAAGGAGTTCAACAAACGGCCCAAGAGCTGGGAGACCTT
BRA1 Sequence 2        ------------------------------------------------------------

BRA1                   AACAGAAAGATCAGAAGTGAACTCGACTGGACTAGGAGAACACATGGAGACTTCTCATCT
BRA1 Sequence 3        ------------------------------------------------------------
BRA2 sequence 4.1      AATAGACGCATCAGGAAAGAACTCGACTGGACCCGGGCCCAGCATGGGGATTACTCTTCC
BRA1 Sequence 1        ------------------------------------------------------------
BRA2 sequence 5.1      AATAGACGCATCAGGAAAGAACTCGACTGGACCCGGGCCCAGCATGGGGATTACTCTTCC
BRA1 Sequence 2        ------------------------------------------------------------
BRA2 sequence 2.1      AATAGACGCATCAGGAAAGAACTCGACTGGACCCGGGCCCAGCATGGGGATTACTCTTCC
BRA2 sequence 2.2      ------------------------------------------------------------
BRA2 sequence 4.2      ------------------------------------------------------------
BRA2 sequence 5.2      ------------------------------------------------------------
BRA2 sequence 3.2      ------------------------------------------------------------
BRA2 sequence 6.2      ------------------------------------------------------------
BRA1 Sequence 1        ------------------------------------------------------------
BRA2 sequence 1.1      AATAGACGCATCAGGAAAGAACTCGACTGGACCCGGGCCCAGCATGGGGATTACTCTTCC
BRA1 Sequence 4        ------------------------------------------------------------
BRA2 sequence 3.1      AATAGACGCATCAGGAAAGAACTCGACTGGACCCGGGCCCAGCATGGGGATTACTCTTCC
BRA1 Sequence 2        ------------------------------------------------------------

BRA1                   TTCCATAGCTATCGCCGAGAACA---GCGTCTCTGGGGACAATGCTCAGAGAACGTCTAC
BRA1 Sequence 3        ------------------------------------------------------------
BRA2 sequence 4.1      TTCGATGGCTATCGGCGAGACCAAGAGCGTCGCTGGGGACAATGCCCAACAAGCATCGGC
BRA1 Sequence 1        ------------------------------------------------------------
BRA2 sequence 5.1      TTCGATGGCTATCGGCGAGACCAAGAGCGTCGCTGGGGACAATGCCCAACAAGCATCGGC
BRA1 Sequence 2        ------------------------------------------------------------
BRA2 sequence 2.1      TTCGATGGCTATCGGCGAGACCAAGAGCGTCGCTGGGGACAATGCCCAACAAGCATCGGC
BRA2 sequence 2.2      ------------------------------------------------------------
BRA2 sequence 4.2      ------------------------------------------------------------
BRA2 sequence 5.2      ------------------------------------------------------------
BRA2 sequence 3.2      ------------------------------------------------------------
BRA2 sequence 6.2      ------------------------------------------------------------
BRA1 Sequence 1        ------------------------------------------------------------
BRA2 sequence 1.1      TTCGATGGCTATCGGCGAGACCAAGAGCGTCGCTGGGGACAATGCCCAACAAGCATCGGC
BRA1 Sequence 4        ------------------------------------------------------------
BRA2 sequence 3.1      TTCGATGGCTATCGGCGAGACCAAGAGCGTCGCTGGGGACAATGCCCAACAAGCATCGGC
BRA1 Sequence 2        ------------------------------------------------------------

BRA1                   AAGTCTGAAGATCCAAAGCGAAGGAGGAAACATCTATGACTGTTGCATCAAAGCCCAAGA
BRA1 Sequence 3        ------------------------------------------------------------
BRA2 sequence 4.1      AAACCTGGAGATTCGAAGCGAAGGAGGAAACATCTATGACTGTTGCATTAAAGCTCAGGA
```

```
BRA1 Sequence 1     ------------------------------------------------------------
BRA2 sequence 5.1   AAACCTGGAGATTCGAAGCGAAGGAGGAAACATCTATGACTGTTGCATTAAAGCTCAGGA
BRA1 Sequence 2     ------------------------------------------------------------
BRA2 sequence 2.1   AAACCTGGAGATTCGAAGCGAAGGAGGAAACATCTATGACTGTTGCATTAAAGCTCAGGA
BRA2 sequence 2.2   ------------------------------------------------------------
BRA2 sequence 4.2   ------------------------------------------------------------
BRA2 sequence 5.2   ------------------------------------------------------------
BRA2 sequence 3.2   ------------------------------------------------------------
BRA2 sequence 6.2   ------------------------------------------------------------
BRA1 Sequence 1     ------------------------------------------------------------
BRA2 sequence 1.1   AAACCTGGAGATTCGAAGCGAAGGAGGAAACATCTATGACTGTTGCATTAAAGCTCAGGA
BRA1 Sequence 4     ------------------------------------------------------------
BRA2 sequence 3.1   AAACCTGGAGATTCGAAGCGAAGGAGGAAACATCTATGACTGTTGCATTAAAGCTCAGGA
BRA1 Sequence 2     ------------------------------------------------------------

BRA1                GGGGACTCTTGCTATTCCTTGCTGCGGGTTCCCACTATGGCTATTCTGGGGACTCATAAT
BRA1 Sequence 3     ------------------------------------------------------------
BRA2 sequence 4.1   GGGGACTCTTGCTATTCCCTGCTGTGGGTTTCCACTGTGGTTATTCTGGGGATTTGTGAT
BRA1 Sequence 1     ------------------------------------------------------------
BRA2 sequence 5.1   GGGGACTCTTGCTATTCCCTGCTGTGGGTTTCCACTGTGGTTATTCTGGGGATTTGTGAT
BRA1 Sequence 2     ------------------------------------------------------------
BRA2 sequence 2.1   GGGGACTCTTGCTATTCCCTGCTGTGGGTTTCCACTGTGGTTATTCTGGGGATTTGTGAT
BRA2 sequence 2.2   ------------------------------------------------------------
BRA2 sequence 4.2   ------------------------------------------------------------
BRA2 sequence 5.2   ------------------------------------------------------------
BRA2 sequence 3.2   ------------------------------------------------------------
BRA2 sequence 6.2   ------------------------------------------------------------
BRA1 Sequence 1     ------------------------------------------------------------
BRA2 sequence 1.1   GGGGACTCTTGCTATTCCCTGCTGTGGGTTTCCACTGTGGTTATTCTGGGGATTTGTGAT
BRA1 Sequence 4     ------------------------------------------------------------
BRA2 sequence 3.1   GGGGACTCTTGCTATTCCCTGCT-------------------------------------
BRA1 Sequence 2     ------------------------------------------------------------

BRA1                CATAATGGGACGTTTGTTCGGCTATGGGTTGCGAGGGATTGCTATGATACTGTCCTATTG
BRA1 Sequence 3     ------------------------------------------------------------
BRA2 sequence 4.1   CATAATAGGGCGATTATTAGGCTATGGACTGCGAGGCGTTGCTATGATACTGCTCTATTG
BRA1 Sequence 1     ------------------------------------------------------------
BRA2 sequence 5.1   CATAATAGGGCGATTATTAGGCTATGGACTGCG---------------------------
BRA1 Sequence 2     ------------------------------------------------------------
BRA2 sequence 2.1   CATAATAGGGCGATTATTAGGCTATGGACTGCGAGGC-----------------------
BRA2 sequence 2.2   ------------------------------------------------------------
BRA2 sequence 4.2   ------------------------------------------------------------
BRA2 sequence 5.2   ------------------------------------------------------------
BRA2 sequence 3.2   ------------------------------------------------------------
BRA2 sequence 6.2   ------------------------------------------------------------
BRA1 Sequence 1     ------------------------------------------------------------
BRA2 sequence 1.1   CATAATAGGGCGATTATTAGGCTATGGACTGCGRGGCRTYGCTATGATACTGYTCTATTG
BRA1 Sequence 4     ------------------------------------------------------------
BRA2 sequence 3.1   ------------------------------------------------------------
BRA1 Sequence 2     ------------------------------------------------------------

BRA1                TGAAAAAGGATTGACTATGCTATTTATAATGCTAAA
BRA1 Sequence 3     ------------------------------------
BRA2 sequence 4.1   TGGGAAAGGATTAACTATGCTGTTTATTATGTAAAA
BRA1 Sequence 1     ------------------------------------
BRA2 sequence 5.1   ------------------------------------
BRA1 Sequence 2     ------------------------------------
BRA2 sequence 2.1   ------------------------------------
BRA2 sequence 2.2   ------------------------------------
BRA2 sequence 4.2   ------------------------------------
BRA2 sequence 5.2   ------------------------------------
BRA2 sequence 3.2   ------------------------------------
BRA2 sequence 6.2   ------------------------------------
BRA1 Sequence 1     ------------------------------------
BRA2 sequence 1.1   TGGGAAAGGATTAACTATGCTGTTTATTA-------
BRA1 Sequence 4     ------------------------------------
BRA2 sequence 3.1   ------------------------------------
BRA1 Sequence 2     ------------------------------------
```

Supplementary Figure S2: Alignment of nucleotide sequences in EIAV LTR using MUSCLE. U3 is in black, R in red, and U5 in blue. Methylated DNA-binding protein site (MDBP) is in the red box, PEA1/AP-1 site in pink, transactivation response element (TAR) is in the green box, initiation site of transcription is in gray, and PU.1 binding sites is in yellow.

```
BRA1     TGTGGGGTTAATAT-AAAATGGCTGTAGAAAAGAAAGTTGCTGATGATTATAAGTA--------------AATATGCTGA
BRA2     ............G.-....A.A.....-..G.........................--------------..........
Liaoning ......A......C-..G..TCT.A..---------------AG.........-----------------..GT......
KY411969 ......A.......-..G..TCT.A..---------------AG.........-----------------..GT......
KY411970 ......A.......-..G..TCT.A..---------------AG.........-----------------..GT......
KY411971 ......A.......-..G..TCT.A..---------------AG.........-----------------..GT......
KY411972 ......A.......-..G..TCT.A..---------------AG.........-----------------..GT......
KY411973 ......A.......-..G..TCT.A..---------------AG.........-----------------..GT......
KY411974 ......A.......-..G..TCT.A..---------------AG.........-----------------..GT......
KY411975 ......A.......-..G..TCT.A..---------------AG.........-----------------..GT......
KY411976 ......A.......-..G..TCT.A..---------------AG.........-----------------..GT......
KY412097 ......A.......-..G..TCT.A..---------------AG.........-----------------..GT......
KY412098 ......A.......-..G..TCT.A..---------------AG.........-----------------..GT......
DLV9-7   ......A.......-..G..TCT.A..---------------AG...A...G.AAGTTGCTGATGCTCTC..GT......
DV10-3   ......A.......-..G..TCT.A..---------------AG....A..G.-----------------..GT......
IRE_F2   ......A....----------ATAA.G---------------AG.AC..G...AA.GA------------..CT......
IRE_F3   ......A....----------ATAA.G---------------AG.AC..G...AA.GA------------..CT......
IRE_F4   ......A....----------ATAA.G---------------AG.AC..G...AA.GA------------..CT......
IRE_H3   ......A....----------ATAA.G---------------AG.AC..G...AA.GA------------..CT......
Wyoming  .........TT...G.GGGGTTT.A..---------------A...........AGTAAAAAG------A..GT......
Miyazaki ....A.A..---------------A..---------------TG.AT..G..TA----------------..AC......

BRA1     TGCTCT-CATAACCGCGTGTAACCTAGAAG-CTA-GCTCATGTTGCTAGGCAACTAAACTGTAATAACCT-GTTAGTTCC
BRA2     ......-......................-A...-.......A..A..A.........A...........-.........
Liaoning ......-.......TTA.......C.A...A...-...........C...............G.......--..T.....
KY411969 ......-.......TTA.......C.A...A...-...........C.......................--..T.....
KY411970 ......-.......TTA.......C.A...A...-...........C.......................--..T.....
KY411971 ......-.......TTA.......C.A...A...-...........C.......................--..T.....
KY411972 ......-.......TTA.......C.A...A...-...........C.......................--..T.....
KY411973 ......-.......TTA.......C.A...A...-...........C.......................--..T.....
KY411974 ......-.......TTA.......C.A...A...-...........C.......................--..T.....
KY411975 ......-.......TTA.......C.A...A...-...........C.......................--..T.....
KY411976 ......-.......TTA.......C.A...A...-.......C...C.......................--..T.....
KY412097 ......-.......TTA.......C.A...A...-........C..C...............G.......--..T.....
KY412098 ......-.......TTA.......C.A...A...-........C..C..............CG..C....--..T.....
DLV9-7   ......-.......TTA..-----------A...-...........C........G......G.......--..T.....
DV10-3   ......-.......TTA.......C.AG..A...-...........C...............G.......--..T.....
IRE_F2   ......-T......T.A.........A...G...-.....G...................A..G......-..A......
IRE_F3   ......-T......T.A.........A...G...-.....G...................A.........-..A......
IRE_F4   ......-T......T.A.........A...G...-.....G...................A..G......-..A......
IRE_H3   ......-T......T.A.........A...G...-.....G...................A..G......-..A......
Wyoming  ......-.......TT..A.....C.A.G.A...-........................C.C.......GCA..T..GA.
Miyazaki ......A.......TT..A.....C.T.TAA...G..AGGC.......A......G....AAT.......--A.......

BRA1     TCATTATTGT---------------------------------------TCCTGTTTTTACACTATATAAGTACATGTAT
BRA2     ..........---------------------------------------.......................G.....G.
Liaoning .......A..----------------TCCGCTTTTGTGACGCGTTAAGT.............G...........T.....
KY411969 .......A..----------------TCCGCTTTTGTGACGCGTTAAGT.............G.........G.T.....
KY411970 .......A..----------------TCCGCTTTTGTGACGCGTTAAGT.............G..C......G.T.....
KY411971 .......A..----------------TCCGCTTTTGTGACGCGTTAAGT.............G.........G.T.....
KY411972 .......A..----------------TCCGCTTTTGTGACGCGTTAAGT.............G.........G.T.....
KY411973 .......A..----------------TCCGCTTTTGTGACGCGTTAAGT.............G.........G.T.....
KY411974 .......A..----------------TCCGCTTTTGTGACGCGTTAAGT.............G.........G.T.....
KY411975 .......A..----------------TCCGCTTTTGTGACGCGTTAAGT.............G.........G.T.....
KY411976 .......A..----------------TCCGCTTTTGTGACGCGTTAAGT.............G.........G.T.....
KY412097 .......A..----------------TCCGCTTTTGTGACGCGTTAAGT.............G...........T.....
KY412098 .......A..----------------TCCGCTTTTGTGACGCGTTAAGT.............G...........T.....
DLV9-7   .......A..TCCGCTTTTGTATAGTTCCGCTTTTGTGACGCGTTAAGT.............G.........G.T.....
DV10-3   .......A..----------------TCCGCTTTTGTGACGCGTTAAGT.............G.........G.T.....
IRE_F2   .......A..----------------TCCGCTTTTGTGACGCGTTAAGT....CAAA.....A........C..T.....
IRE_F3   .......A..----------------TCCGCTTTTGTGACGCGTTAAGT....CAAA.....A........C..T.....
IRE_F4   .......A..----------------TCCGCTTTTGTGACGCGTTAAGT....CAAA.....A........C..T.....
IRE_H3   .......A..----------------TCCGCTTTTGTGACGCGTTAAGT....CAAA.....A........C..T.....
Wyoming  G.G----A..----------------TCCCCATTGGTGACGCGTTAACT.............G.........G.T.....
Miyazaki CT...TAA..----------------TCCGCTTTTGTGACGCGTTAAGT.....A.......A.........G.T.A...
```

```
BRA1      TCTGATAACAAGACACTCAGATTCTGCGGTCTGAGTCCCTTCTCTGCTGGGCCTAATA--AGCCTTGA-AATAA--ATAT
BRA2      .T..T.......................................................C.--......A.G.....--....
Liaoning  .....C.TTTG.........................................T-..CTCT.......GT.....--....
KY411969  .....C.TTTG.........................................T-G.CTCT.......GT.....--....
KY411970  .....C.TTTG.........................................T-G.CTCT.......GT.....--....
KY411971  .....C.TTTG.........................................T-G.CTCT.......GT.....--....
KY411972  .....C.TTTG.........................................T-G.CTCT.......GT.....--....
KY411973  .....C.TTTG.........................................T-G.CCCT.......GT.....--....
KY411974  .....C.TTTG.........................................T-G.CTCT.......GT.....--....
KY411975  .....C.TTTG.........................................T-G.CTCT.......GT.....--....
KY411976  .....C.TTTG.........................................T-G.CTCT.......GT.....--....
KY412097  .....C.TTTG.........................................T-..CTCT.......GT.....--....
KY412098  .....C.TTTG.........................................T-..CTCT.......GT.....--....
DLV9-7    .....C.TTTG.T.G.....................................TA..CTCT.......GT.....--....
DV10-3    .....C.TTTG.........................................TA..CTCT.......GT.....--....
IRE_F2    .....C.CTGG.........................................TA..A---......CTT.....--....
IRE_F3    .....C.CTGG.........................................TA..A---......CTT.....--....
IRE_F4    .....C.CTGG.........................................TA..A---......CTT.....--....
IRE_H3    .....C.CTGG.........................................TA..A---......CTT.....--....
Wyoming   .....C..TTG.G-......................................TG..A.--G.....TGT.....--....
Miyazaki  ....TC.TTTG.G.......................................TA...---........C.....AT....
```

```
BRA1      AATTCTCTA-CTCAGTCCCTTGTCTCAACTTGT--TGGTTTTAA-GATCCTACA
BRA2      ........GA...............A.......-T..A......-.........
Liaoning  ........G-..........GT...T.GTC...CT..T...C..-.G..TA...
KY411969  ........G-T.........GT...T.GT....CT..T...C..-....TA...
KY411970  ........G-T.........GT...T.GT....CT..T...C..-....TA...
KY411971  ........G-T.........GT...T.GT....CT..T...C..-....TA...
KY411972  ........G-T.........GT...T.GT....CT..T...C..-....TA...
KY411973  ........G-T.........GT...T.GT....CT..T...C..-....TA...
KY411974  ........G-T.........GT...T.GT....CT..T...C..-....TA...
KY411975  ........G-T.........GT...T.GT....CT..T...C..-....TA...
KY411976  ........G-T.........GT...T.GT....CT..T...C..-....TA...
KY412097  .......CG-..........GT...T.GTC...CT..T...C..-....TA...
KY412098  ........G-..........GT...T.GTC...CT..T...C..-....TA...
DLV9-7    ........G-..A.......GT...T.GT....CT..T...C..-....TA...
DV10-3    ........G-..A.......GT...T.GT....CT..T...C..-....TA...
IRE_F2    .........-......T...GTGT.......A.CTCTTGG....-.........
IRE_F3    .........-......T...GTGT.......A.CTCTTGG....-.........
IRE_F4    .........-......T...GTGT.......A.CTCTTGG....-.........
IRE_H3    .........-......T...GTGT.......A.CTCTTGG....-.........
Wyoming   .........-..........GTCTCT.GT....C---TG..CG.-.........
Miyazaki  ........G-T.........GTGT.........TT..C..AC.GCA.C..A...
```

Supplementary Figure S3: Alignment of EIAV gag polyprotein. The proteins p15, p26, p11 and p9 are separated by blue arrows. Amino acid variations between BRA1 and BRA2 are in yellow. A leucine-rich-type nuclear export signal is in the green box (p15), major homology region is in the red box (p26), two zinc binding domains are in the blue boxes (p11), and YPDL late domain is in the orange box (p9).

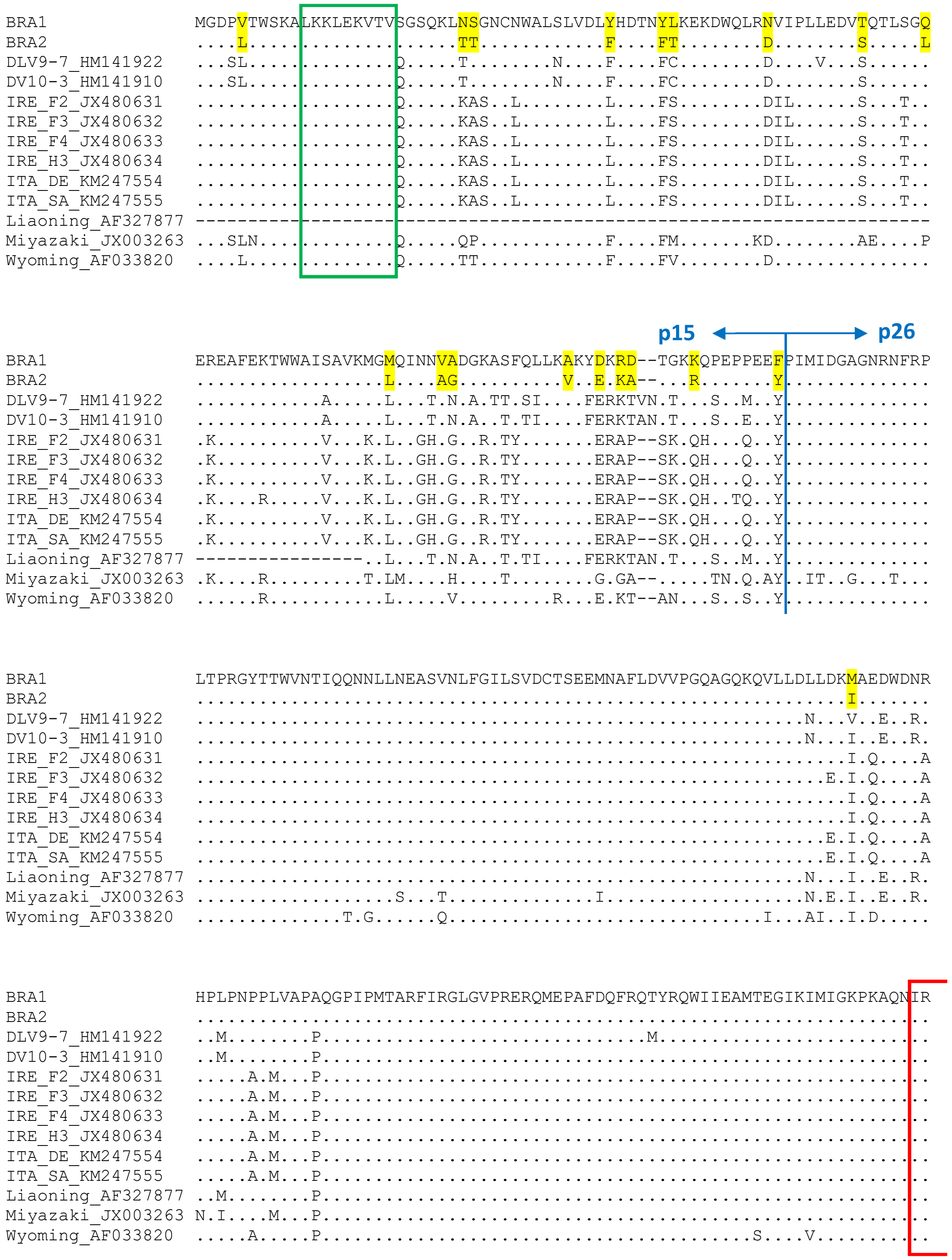

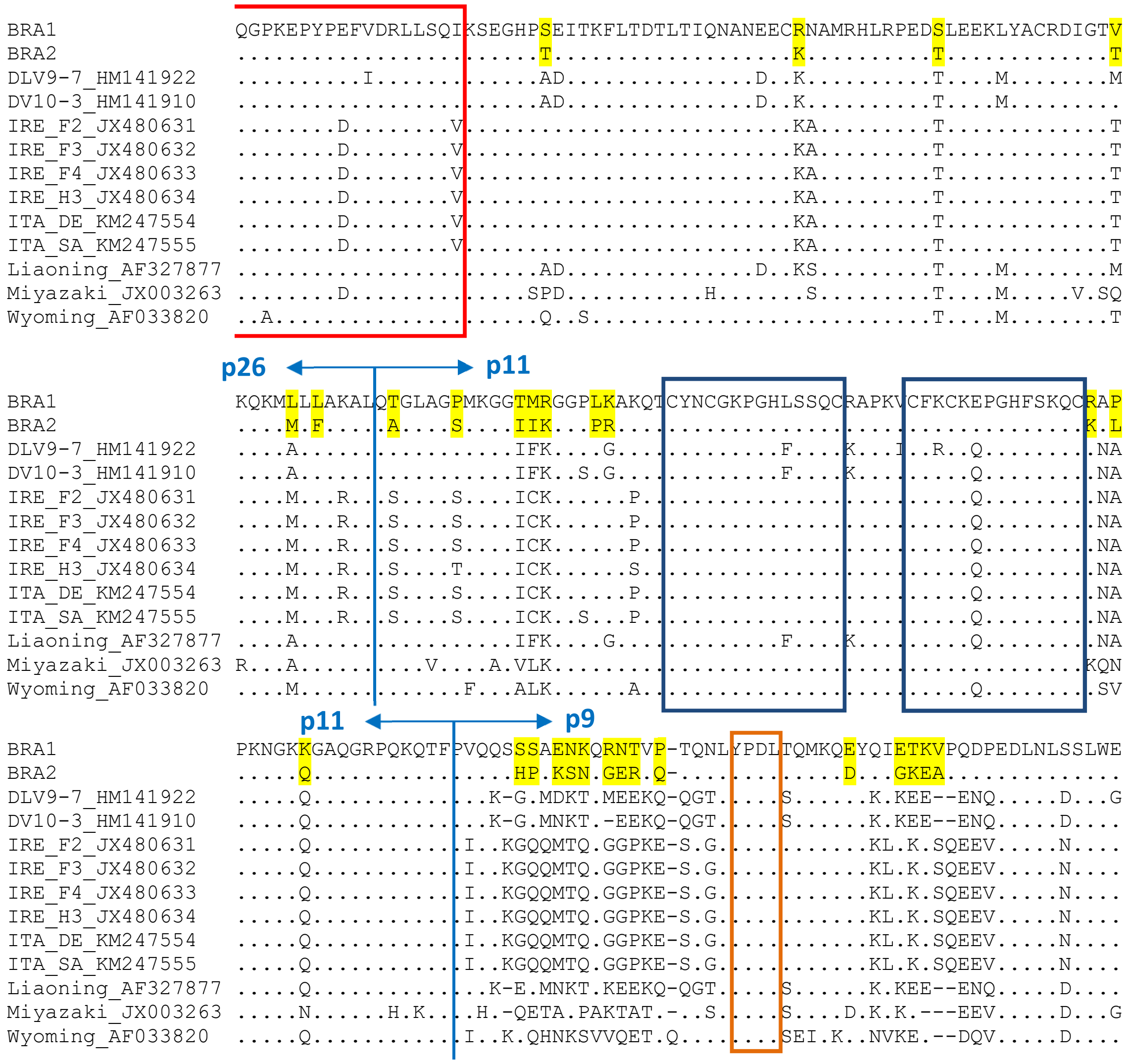

BRA1 QGPKEPYPEFVDRLLSQIKSEGHPSEITKFLTDTLTIQNANEECRNAMRHLRPEDSLEEKLYACRDIGTV
BRA2 ........................T...................K..........T.............T
DLV9-7_HM141922 ..........I............AD................D..K..........T....M........M
DV10-3_HM141910 .......................AD................D..K..........T....M.........
IRE_F2_JX480631 ........D........V..........................KA.........T.............T
IRE_F3_JX480632 ........D........V..........................KA.........T.............T
IRE_F4_JX480633 ........D........V..........................KA.........T.............T
IRE_H3_JX480634 ........D........V..........................KA.........T.............T
ITA_DE_KM247554 ........D........V..........................KA.........T.............T
ITA_SA_KM247555 ........D........V..........................KA.........T.............T
Liaoning_AF327877 .......................AD................D..KS.........T....M........M
Miyazaki_JX003263 ........D.............SPD............H.......S.........T....M.....V.SQ
Wyoming_AF033820 ..A.....................Q..S...........................T....M........T
p26
p11
BRA1 KQKMLLLAKALQTGLAGPMKGGTMRGGPLKAKQTCYNCGKPGHLSSQCRAPKVCFKCKEPGHFSKQCRAP
BRA2 ....M.F.....A....S....IIK...PR.....................................K.L
DLV9-7_HM141922 ....A.................IFK....G.............F....K...I..R..Q.........NA
DV10-3_HM141910 ....A.................IFK..S.G.............F....K.........Q.........NA
IRE_F2_JX480631 ....M...R...S....S....ICK......P..........................Q.........NA
IRE_F3_JX480632 ....M...R...S....S....ICK......P..........................Q.........NA
IRE_F4_JX480633 ....M...R...S....S....ICK......P..........................Q.........NA
IRE_H3_JX480634 ....M...R...S....T....ICK......S..........................Q.........NA
ITA_DE_KM247554 ....M...R...S....S....ICK......P..........................Q.........NA
ITA_SA_KM247555 ....M...R...S....S....ICK..S...P..........................Q.........NA
Liaoning_AF327877 ....A.................IFK....G.............F....K.........Q.........NA
Miyazaki_JX003263 R...A..........V....A.VLK..KQN
Wyoming_AF033820 ....M.............F...ALK......A..........................Q.........SV
p11
p9
BRA1 PKNGKKGAQGRPQKQTFPVQQSSSAENKQRNTVP-TQNLYPDLTQMKQEYQIETKVPQDPEDLNLSSLWE
BRA2 .....Q................HP.KSN.GER.Q-.............D...GKEA..............
DLV9-7_HM141922 .....Q..............K-G.MDKT.MEEKQ-QGT.....S......K.KEE--ENQ.....D...G
DV10-3_HM141910 .....Q..............K-G.MNKT.-EEKQ-QGT.....S......K.KEE--ENQ.....D....
IRE_F2_JX480631 .....Q............I..KGQQMTQ.GGPKE-S.G............KL.K.SQEEV.....N....
IRE_F3_JX480632 .....Q............I..KGQQMTQ.GGPKE-S.G............KL.K.SQEEV.....N....
IRE_F4_JX480633 .....Q............I..KGQQMTQ.GGPKE-S.G............KL.K.SQEEV.....N....
IRE_H3_JX480634 .....Q............I..KGQQMTQ.GGPKE-S.G............KL.K.SQEEV.....N....
ITA_DE_KM247554 .....Q............I..KGQQMTQ.GGPKE-S.G............KL.K.SQEEV.....N....
ITA_SA_KM247555 .....Q............I..KGQQMTQ.GGPKE-S.G............KL.K.SQEEV.....N....
Liaoning_AF327877 .....Q..............K-E.MNKT.KEEKQ-QGT.....S......K.KEE--ENQ.....D....
Miyazaki_JX003263 .....N......H.K....H.-QETA.PAKTAT.-..S.....S....D.K.K.---EEV.....D...G
Wyoming_AF033820 .....Q............I..K.QHNKSVVQET.Q........SEI.K..NVKE.--DQV.....D....

Supplementary Figure S4: Alignment of EIAV pol polyprotein. In PR, the catalytic motif DTG is in the red box, substrate-binding sites are in cyan and active sites flap are in the box orange. In RT, DIGD motif is in the purple box and DD motif is in the gray box. In RNase H, invariant residues are in cyan and the glycine-rich motif is in the dark green box. In DU, the conserved motifs MI-MV are in the dark blue boxes, with the conserved residue D72 in MIII in cyan. In IN, the zinc finger-binding motif (H12, H16, C40, C43) are in green, hydrophobic core residues (I5, A8, L22, A33 and I36) are in pink, and central catalytic core residues (D64, D116 and E152) are in blue.

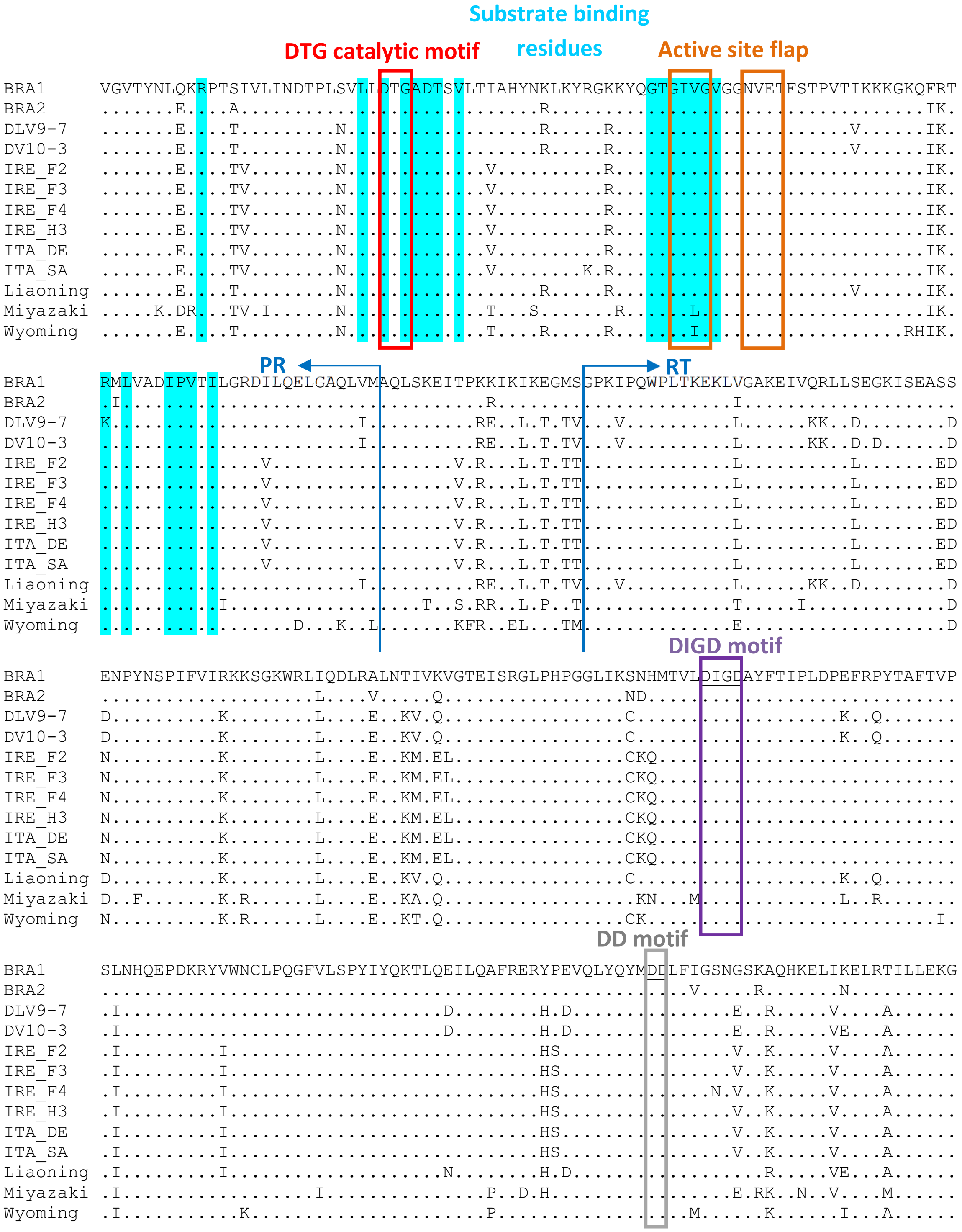

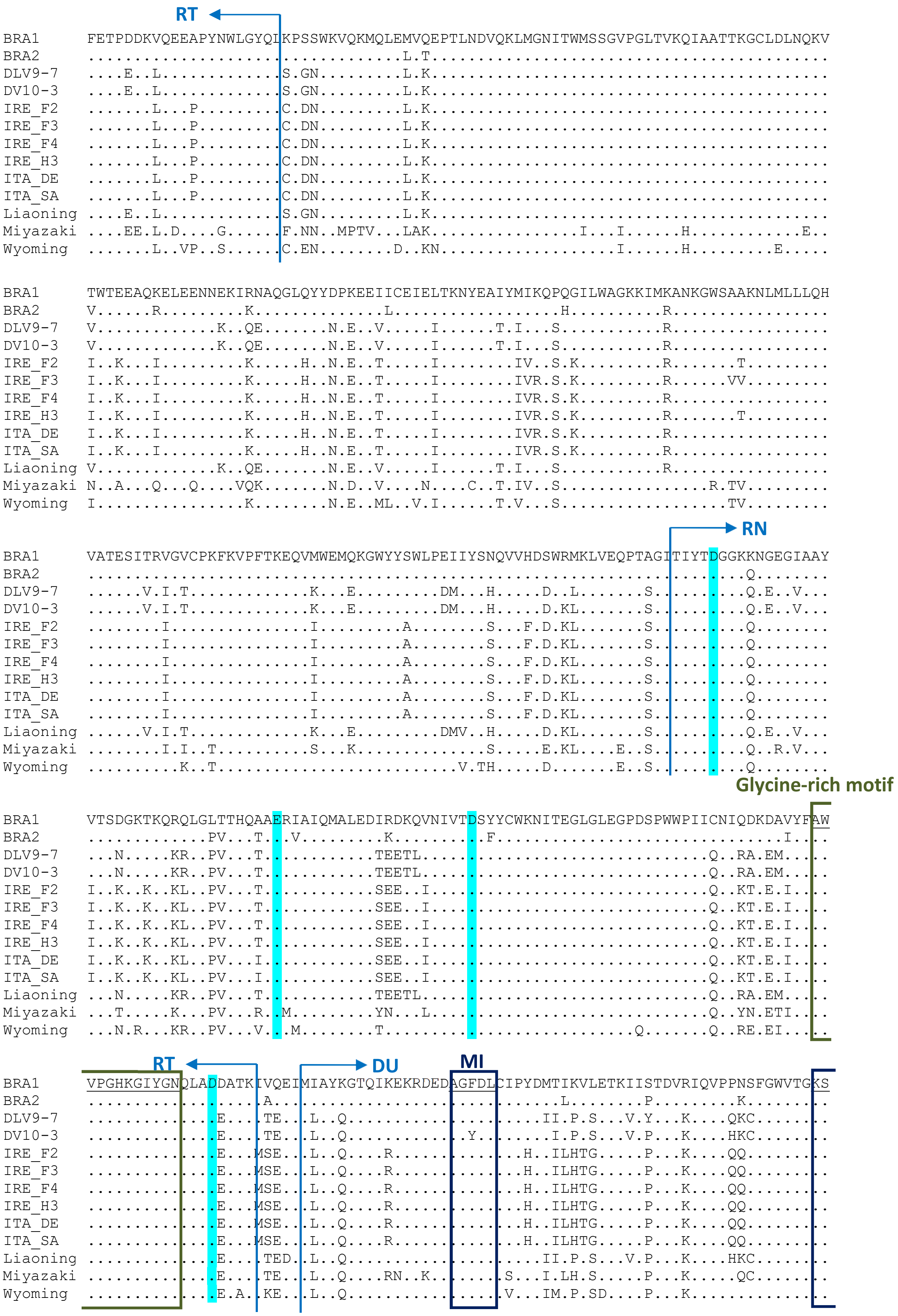
RT
RN
Glycine-rich motif
RT
DU
MI
BRA1
BRA2
DLV9-7
DV10-3
IRE_F2
IRE_F3
IRE_F4
IRE_H3
ITA_DE
ITA_SA
Liaoning
Miyazaki
Wyoming

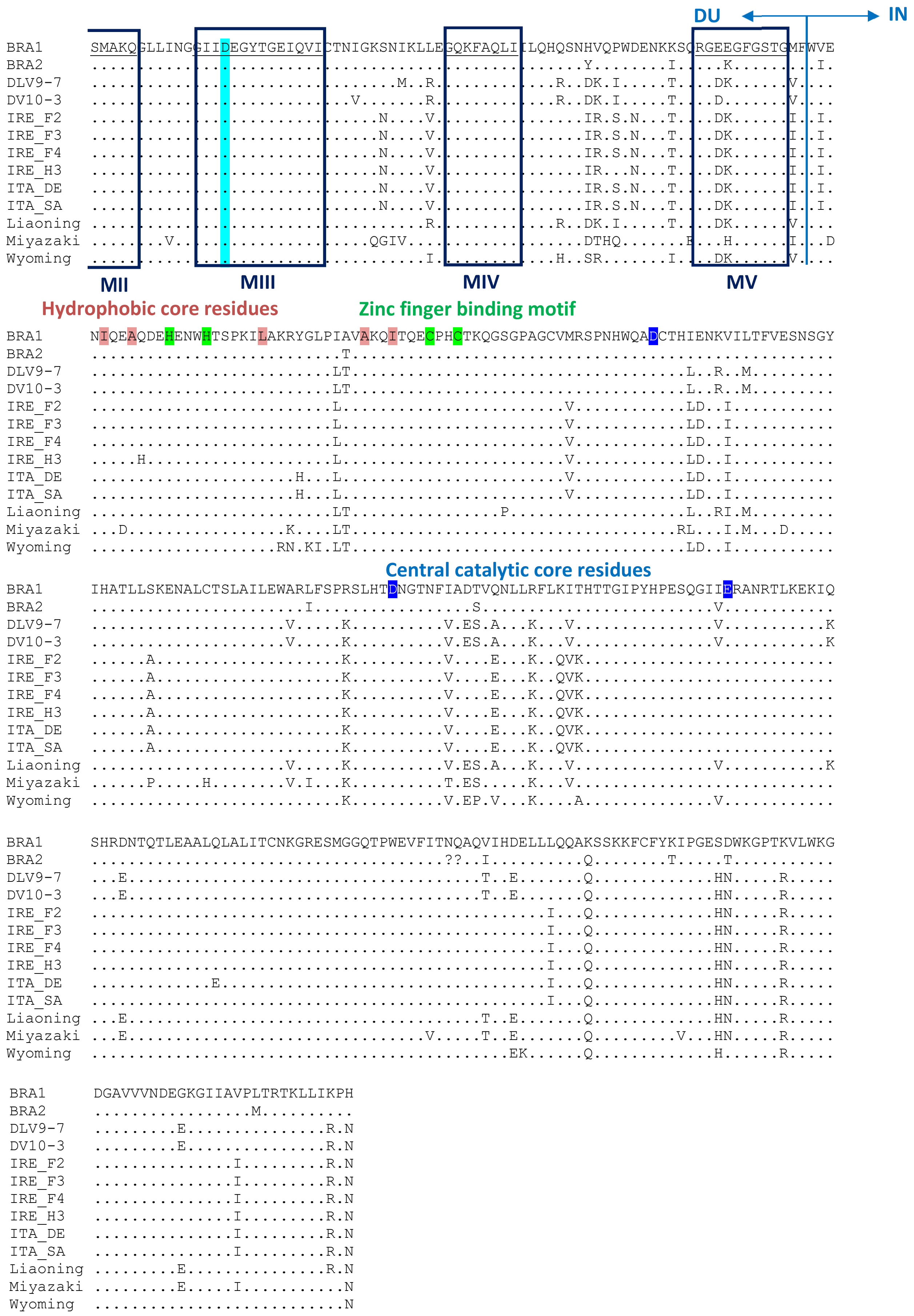
DU
IN
MII
MIII
MIV
MV
Hydrophobic core residues
Zinc finger binding motif
Central catalytic core residues

Supplementary Figure S5: Alignment of EIAV env. The surface protein and transmembrane protein were aligned using MUSCLE. Hipervariable regions are marked with colors: yellow - V1, green - V2, cyan - V3, blue - V4, orange - V5, purple - V6, dark green - V7, and red - V8. While cysteine residues are with red letters.

```
BRA1                 MVSIAFYGGI PGGISTPIIQ S--SEEASHT KGD-TIFKPY CSDEKSNRNK TMAEERD---
BRA2                 .......... ...V.A..V. .--P...TC. ...-...... .YNDN..KD. K...G..---
DLV9-7_HM141922      .......... ..E.....T. Q--T.STDTQ ...HMVYQ.. .YNDSHKEE- -...A..---
DV10-3_HM141910      .......... ...V....T. Q--T.STDTQ ...HMVYQ.. .YNDSHKAE- -...A..---
IRE_F2_JX480631      .......... ...VQ...V. QSEP.SKGSE .R.YE..Q.. .Y.SSYKTK- -...G..---
IRE_F3_JX480632      .......... ...VQ...V. QSEP.SKGSE .R.YE..Q.. .Y.SSYKTK- -...G..---
IRE_F4_JX480633      .......... ...VQ...V. QSEP.SKGSE .R.YE..Q.. .Y.SSYKTK- -...G..---
IRE_H3_JX480634      .......... ...VQ...V. QSEP.SKGSE .R.YE..Q.. .Y.SSYKTK- -...G..---
ITA_DE_KM247554      .......... ...VQ...V. QSEP.SKGSE .R.YE..Q.. .Y.SSYKTE- -...G..---
ITA_SA_KM247555      .......... ...VQ...V. QSEP.SKGSE .R.YK..Q.. .Y.SSYKTE- -...G..---
Liaoning_AF327877    .......... ........T. Q--TKSTDTQ ...HMVYQ.. .YNDSHKAE- -...A..---
Miyazaki_JX003263    .......... ........SS E--..Q.DTD .RGPMV.Q.. .YNGSNK.R- -....GKQIP
Wyoming_AF033820     .......... ........T. Q--..KSKCE EN--.M.Q.. .YNND.KNS- -...SKE---

BRA1                 SRERREAYLK EDAEENAKKR NNDWWKIGMF MLCLMGATGG MLWWYEDVAH PQYIGLITVG
BRA2                 P.D...MN.. ..P..K.R.. .......... ......T... I.....G... .R........
DLV9-7_HM141922      T.YQE.MNQ. .EK.D.--.. R.N....... L...L.T... F.....RQQ. SY....V.I.
DV10-3_HM141910      T.YQE.MNR. ..K.D---.. R.N....... L...L.T... F.....GQH. SH....V.I.
IRE_F2_JX480631      ..YQE.MIP. .ESKGKEE.G R......... ....L..A.. I.....GTPD VH....VA..
IRE_F3_JX480632      ..YQE.MIP. .ESKGKEE.G R......... ....L..A.. I.....GTPD VH....VA..
IRE_F4_JX480633      ..YQE.MIP. .ESKGKEE.G R......... ....L..A.. I.....GAPD VH....VA..
IRE_H3_JX480634      ..YQE.MIP. .ESKGKEE.G RK........ ....L..A.. I.....GTPD VH....VA..
ITA_DE_KM247554      ..YQE.MIP. .ESKGKEE.G R......... ....L..A.. I.....GAPD VH....VA..
ITA_SA_KM247555      ..YQE.MIP. .ESKGKEE.G R......... ....L..A.. I.....GAPD VH....VA..
Liaoning_AF327877    T.YQE.MNR. .EK.D.--.. R.N....... L...L.T... F.....GQK. SH....V.I.
Miyazaki_JX003263    PE..E.LFQG .ELKK---E. R......... L...L.T... F.....GQE. SH...PVA..
Wyoming_AF033820     A.DQ-.MN.. .ESK.E--.. R......... L...A.T... I.....GLPQ QH....VAI.

BRA1                 GKLEGSGMTS AIECWGKFPG CRPFDNYFSY MTNRRIHVQN NT-VSLLESY HREITYIYKT
BRA2                 ....E..... ......T... ....T..... E...T.QET. D.-AT..... N??-------
DLV9-7_HM141922      .R.N...... ......S... ....T..... E...TVSRD. ..-AT..DA. Q..V.N..R.
DV10-3_HM141910      .R.N...... ......S... ....T..... E...TVSRD. ..-AT..DA. Q....N..R.
IRE_F2_JX480631      .R.N.....N ......T... ....T..... K...S..AD. ..-AT..NA. ..........
IRE_F3_JX480632      .R.N.....N ......T... ....T..... K...S..AD. ..-AT..NA. ..........
IRE_F4_JX480633      .R.N.....N ......T... ....T..... K...S..AD. ..-AT..NA. ...V......
IRE_H3_JX480634      .R.N.....N ......T... ....T...R. K...S..AD. ..-AT..NA. ...V......
ITA_DE_KM247554      .R.N.....N ......T... ....T..... K...S..AD. ..-AT..NA. ...V......
ITA_SA_KM247555      .R.N.....N ......T... ....T..... K...S..AD. ..-AT..NA. ...V......
Liaoning_AF327877    .R.N...... ......S... ....T..... E...T.SRD. ..-AT..DA. Q..V.N..R.
Miyazaki_JX003263    .G.N...... .....SS... ....N..... E..KT.NID. ..AAT..QA. ..K.G.L...
Wyoming_AF033820     .R.N...QSN ......S... ....Q..... E...SM.MD. ..-AT...A. .....F...S

BRA1                 MCTDSDHCQE YECRNVQLTG NGLKVNISYN TTLDTP---Y WNFKWLVCNQ TENQKAILIP
BRA2                 -........? ...ER.R.N. -NFSDR..VE DNNNST---. .D.T..L... ...K.T..V.
DLV9-7_HM141922      S.V......K .K.KQ...E- -KNSN..IM. NCSNNSCEEF .G.S..E... ..SAI...V.
DV10-3_HM141910      S.V....... .K.KQ...K- -ENSS..IM. NCSSNSCEEF RG.S..E... ...AIT..V.
IRE_F2_JX480631      S.V....... .V.QV.DM.- -DKNNTLTIS I.GNGT--D. .G.Q..E... ...A.T.VV.
IRE_F3_JX480632      S.V....... .V.QV.DM.- -DKNNTLTIS I.GNGT--D. .G.Q..E... ...A.T.VV.
IRE_F4_JX480633      S.V....... .V.Q.ISM.- -NKGSTLTI. I.G.GT--D. .G.Q..E... ...A.TMVV.
IRE_H3_JX480634      S.V....... .V.Q.ISM.- -DNGN..II- -.GNGT--D. .G.Q..E... ...A.T.VV.
ITA_DE_KM247554      S.V....... .V.QA.NM.- -DGGNTLTIS I.GNRT--D. .G.R..E... ...A.T.VV.
ITA_SA_KM247555      S.V....... .V.QV.NM.- -DRGNTLTIS I.GNGT--D. .G.Q..E... ...A.T.VV.
Liaoning_AF327877    S.V....... .K.KQ...R- -ENSS..IM. NCSNNSCEEF .G.S..E... ...AIT..V.
Miyazaki_JX003263    S.V....... .T.QKFDADK IDNGST-TI. I.IIS.---- --.NL.E..L ..KA.T.VV.
Wyoming_AF033820     S......... .Q.KK.N.NS SDSSNSVRVE DVMN.A--E. .G....E... ...F.T..V.

BRA1                 EEEMVQ-NDN NTWSPRGCNS TWARVKHCPA DLLYGIHPIR LCVQPPFFLV KNNDGNFTNG
BRA2                 ....I.Y... R..A.K...D ...K.....M .....L.... .......... RKE.NHS---
DLV9-7_HM141922      .V..Q.--SK ...I.KR.KK ...K.....M ......NR.. M........F .QD.TSNNT-
DV10-3_HM141910      DI..QE-S.. ...I.KR..E .R.......M ......NR.. M........F .Q..TSNNT-
IRE_F2_JX480631      IN...RIDNS SS.V.K...E ......Q..V .......... .......... ..DSS.DNSS
IRE_F3_JX480632      IN...RIDNS SS.V.K...E ......Q..V .......... .......... ..DSS.DNSS
IRE_F4_JX480633      IN.....I-.. SS.V.K...E ......Q..V .......... .......... ..DSS.DNSS
IRE_H3_JX480634      IN.....I-.. SS.V.K...E ......Q..V .......... .......... ..DSS.DNSS
```

```
ITA_DE_KM247554      IN....IDNS SS.V.K...E ...K..Q..V .......... .......... ..DSS.SNSS
ITA_SA_KM247555      MN....IDNS SS.V.K...E ......Q..V .......... .......... ..DSS.GISS
Liaoning_AF327877    .V..Q?-S.. ...I.KR..E .........M ......NR.. M........F .Q..TSNNT-
Miyazaki_JX003263    .Q...NI--S .S.Y.K.Y.E ...K.....I .......... .......... RK.NDTN---
Wyoming_AF033820     .N...NI..T D..I.K...E ......R..I .I........ .......... QEKGIAD.--

BRA1                 NHTLSNCGPQ ISLGILDENK AVTR-EV-NC TVAKKVFHFN D-YSGQFISP IFYQCNLS-L
BRA2                 -R........ .......S.. .AVK-DA-S. Q.E..T.Q.S KGH...L.V. ...K.TITN.
DLV9-7_HM141922      -GI......L VF....ED.. .AIQ-NG-S. .LHRTNIERP .-...FYQV. ...I...TG.
DV10-3_HM141910      -GI......L VF....ED.. .AVQ-NG-S. .LHRTNIKRP .-...FYQV. ...I...TG.
IRE_F2_JX480631      .S.......S .F....ED.. ..VT-.G-.. .LHRRNINRP L-.Y..Y.V. ...K.SFN-Y
IRE_F3_JX480632      .S.......S .F....ED.. ..VT-.G-.. .LHRRNINRP L-.Y..Y.V. ...K.SFN-Y
IRE_F4_JX480633      .S.......S .F....ED.. ..VT-KG-.. .LH..NINRP L-.Y..Y.V. ...K..F.-Y
IRE_H3_JX480634      .S.......S .F....ED.. ..VT-.G-.. .LHRRNINRP L-.Y..Y.V. ...N..F.-Y
ITA_DE_KM247554      .S.......S .F....ED.. ..VT-.G-S. .LHRTSIYRS L-.Y..Y.V. ...N.SFN-Y
ITA_SA_KM247555      .S.......S .F....ED.. ..VT-.G-.. .LHRTNINRP L-.Y..Y.V. ...N..F.-Y
Liaoning_AF327877    -SI......L VF....ED.. .AIQ-NG-S. .LHRTNIKRP .-...FYQV. ...I...TG.
Miyazaki_JX003263    -......... VF..V..D.. T.VK-SEEK. KIIRFNIKRQ .-.F..Y.T. .V.NYT.N-I
Wyoming_AF033820     -SRIG....T .F..V.ED.. G.V.GDYTA. N.RRLNINRK .-.T.IYQV. ...T.TFTNI

BRA1                 TPCN-GTNSP VSIIRYDEDT VEYLIRNTVE YLICKA---- ---KNITEKN F---SCVVQT
BRA2                 .R.Y-NNS.T I.......KN .Q-------- ..L..T---- ---N.TDAG. .---......
DLV9-7_HM141922      QS..--NG.I I...M.ESNN .Q-------- ..L.NT---- ---S.TNST. NANV.....S
DV10-3_HM141910      QS..--NG.I I...M.ESNN .Q-------- ..L.NT---- ---S.TNST. NANV.....S
IRE_F2_JX480631      .S..--DD.L I.V...EP.N .Q-------- ..L.NI---- ---N.TSDT. NA--..I...
IRE_F3_JX480632      .S..--ND.L I.V.M.EP.N .Q-------- ..L.NINGTN NTSN.TS.I. NA--..I...
IRE_F4_JX480633      .S.D--NDPL I.V.M.EP.N .Q-------- ..L.NINGTN NTSN.TRNI. NA--..I...
IRE_H3_JX480634      .S.D--NDPL I.V.M.EP.N .Q-------- ..L.NINGTN NTSN.TSKI. NA--..I...
ITA_DE_KM247554      .S..--ND.L I.V.MHEPGN LQ-------- ..L.NI---- ---N.TSKI. NA--..I...
ITA_SA_KM247555      .S..--ND.L I.V.M.EP.N .Q-------- ..L.NI---- ---N.TSDI. NA--..I...
Liaoning_AF327877    QS..--NG.I I...MSESNN .Q-------- ..L.NT---- ---S.TNST. NATV.....S
Miyazaki_JX003263    SR..QKN.KS ..V.M.ESGN IQ-------- ..L.NVNNEN NNTNITDAIK NY--I.IA.S
Wyoming_AF033820     .S..--NEPI I.V.M.ETNQ .Q-------- ..L.NN---- ---N.SNNY. -----....S

BRA1                 FGTIGQAHIE LPRKNKKIIG NQFTQYNCSI NDKTELEKWK LVKSSGITPF PVTSRANTGL
BRA2                 ..K....... ...N..R.RE .K........ .NQ...KE.. .I.G.....I .I..Q.....
DLV9-7_HM141922      ..V.....VA ......RLQS PN.A....T. .N....RQ.Q ...T.....L .IS.T.....
DV10-3_HM141910      ..V.....VA ......RLQS PK.AH..... .N....RQ.Q ...T.....L .IS.T.....
IRE_F2_JX480631      ..I.....L. ......R.AT PN.SH...TV .N...MQE.Q ...T.....I .IS..NEK..
IRE_F3_JX480632      ..I.....L. ......R.TE PN.SH...TV .N...MQE.Q ...T.....I .IS..NEE..
IRE_F4_JX480633      ........L. ......R.AT PN.SH...TV .N...MQE.Q ...T.....I .IS..NEE..
IRE_H3_JX480634      ..I.....L. ......R.AT PN.SH...TV .N...MQE.Q ...T.....I .IS..NEK..
ITA_DE_KM247554      ..I.....L. ......R.AE PG.SH...TV .N...MQE.Q ...T.....I .IS..SEK..
ITA_SA_KM247555      ........L. ......R.AE PD.SH...TV .N...MQE.Q ...T.....I .IS..NEK..
Liaoning_AF327877    ..V.....VA ......RLQS PK.AH...T. .N....RQ.Q ...T.....L .IS.T.....
Miyazaki_JX003263    ..K.....L. ...PT.G.KQ QN..R..... ..QG.MR..T ...T.....L ..S.SD.S..
Wyoming_AF033820     ..V.....L. ...P..R.RN QS.N...... .N.....T.. ...T.....L .IS.E.....

                gp90 ←——|——→ gp45
BRA1                 VRYKRDFGIS AIVAAIVAAT AIAASATMSY IALTETNKMS DVMNHTFVVE NDTIRATEMI
BRA2                 I......... .......... .......... .....AS.V. NA.....E.. .N..H.....
DLV9-7_HM141922      ..H....... ..I....... .......... .....V..LD G.Q....E.. .N..NSM.LT
DV10-3_HM141910      ..H....... ..I....... .......... .....V..LD S.Q....E.. .N..NSM.L.
IRE_F2_JX480631      ..H.....L. .......... .....T.... .....AS.IQ ..V...LN.. .N.LS.....
IRE_F3_JX480632      I.H.....L. .......... .....T.... .....AS.IQ ..V...LN.. .N.LS.....
IRE_F4_JX480633      I.H.....L. .......... .....T.... .....AS.IQ ..V...LN.. .N.LS.....
IRE_H3_JX480634      ..H.....L. .......... .....T.... .....AS.IQ ..V...LN.. .N.LS.....
ITA_DE_KM247554      I.H.....L. .......... .....T.... .....AS.IQ ..V...LN.. .N.LS....M
ITA_SA_KM247555      I.H.....L. .......... .....T.... .....AS.IQ ..V...LN.. .N.LS.....
Liaoning_AF327877    ..H....... ..I....... .......... .....V..LD S.Q....E.. .N..NGL.LV
Miyazaki_JX003263    I......... ..I....... .......... ....Q..HLM ..T....E.. .N..SGM.L.
Wyoming_AF033820     I.H....... .......... .......... V....V..IM E.Q....E.. .S.LNGMDL.

BRA1                 EKQIHILYAM VLQTHADVQL LKERQKIEET FNLIGCVERS HTFCHTGHPW NESWGQLNET
BRA2                 ...M...... I......... .......D.. .......... .......... .D......DS
DLV9-7_HM141922      .E........ .......... ...Q...... ......I... .......... ........DS
DV10-3_HM141910      .E........ .......... ...Q...... ......I... .......... ........DS
IRE_F2_JX480631      .R..N..... I......... ...K.QV... ......I.KT .......... .N...L..DS
IRE_F3_JX480632      .R..N..... I......... ...K.QV... ......I.KT .......... .N...F..DS
IRE_F4_JX480633      .R..N..... I......... ...K.QV... ......I..T .......... .N...L..DS
IRE_H3_JX480634      ...VS..... I......... ...K.QV... ......I.KT .......... .N...L..D.
ITA_DE_KM247554      ....S..... I......... ...K.QV... ......I..T .......... .N...L..DS
```

```
ITA_SA_KM247555     .R.VS..... I......... ...K.QV... ......I..T .......... .N...F..DS
Liaoning_AF327877   .E........ .........S ...Q...... ......I... .......... ........DS
Miyazaki_JX003263   .....M.... I......... ...Q...... ..M...A... .I........ ..T..S..DS
Wyoming_AF033820    .R..K..... I......... .....QV... ......I..T .V........ .M...H...S

BRA1                TEWDDWITKM NRLETEILTT LHAAKNNLEQ AMITFNTPDS IAQFGRNIWS HIANWIPGLG
BRA2                .Q....VN.. .EY.QD.... ....R..... .......... .....K.... ..........
DLV9-7_HM141922     .Q....VD.. EN.NHD.... ..T.R..... S......... .....K.... ..........
DV10-3_HM141910     .Q....VD.. EN.NHD.... ..T.R..... S......... .....K.... ..........
IRE_F2_JX480631     .Q..T.VGE. EKYNQD.... ..I.R..... .......... .....K...D ..........
IRE_F3_JX480632     .Q..T.VGE. EKYNQD.... ..I.R..... .......... .....K...D ..........
IRE_F4_JX480633     .Q..T.VGE. EKYNQD.... ..V.R..... .......... .....K...D ..........
IRE_H3_JX480634     .Q..T.VGE. EKYNQD.... ..V.R..... .......... .....K...D ..........
ITA_DE_KM247554     .Q..T.VGE. EKYNQD.... ..I.R..... .......... .....K...D ..........
ITA_SA_KM247555     .Q..T.VGE. EKYNQD.... ..I.R..... .......... .....K...D ..........
Liaoning_AF327877   .Q....VD.. EN.NHD.... ..T.R..... S......... .....K.... ..........
Miyazaki_JX003263   .Q....V.R. .G..H.V... ....R..... SI.......T .....K.... .V........
Wyoming_AF033820    .Q....VS.. ED.NQ..... ..G.R...A. S......... .....KDL.. ..G.......

BRA1                ASIIKYIIML LLCYVVLTSS PKILRNLLRM MSGAGSSVNR YLKRQHHQKH AWKEDAWDQD
BRA2                .......V.. .......... ........TT .........H F..KK..... ....ES....
DLV9-7_HM141922     .......VL. ..V..L...A .....G..T. ........S. ..RKRY.HR. .SRG.I.A.V
DV10-3_HM141910     .......VL. ..V..L...A .....G..TT .......AS. ..RKRY.HR. .SRG.I.A.V
IRE_F2_JX480631     .......VLF .II......M ..L..H..TT .T..A..AS. ....KL.H.R .SQDGN..R.
IRE_F3_JX480632     .......VLF .II......M ..L..H..TT .T.....AS. ....KL.H.R .SQDGH..R.
IRE_F4_JX480633     .......VLF .II......M ..L..H..TT .T.....AS. ....KL.H.R .SQDGN..R.
IRE_H3_JX480634     .......VLF .II......M ..L..H..TT .T.....AS. ....KL.H.R .SQDGH..R.
ITA_DE_KM247554     .......VLF .II......M ..L..H..TT .T....FASH ....KL.H.R .SQDGH..R.
ITA_SA_KM247555     .......VLF .II......M ..L..H..TT .T.....AS. ....KL.H.R .SQDGH..R.
Liaoning_AF327877   .......VL. ..V..L...A .....G..TT .......AS. ..RKRY.HR. .SRG.I.A.V
Miyazaki_JX003263   ..LT..V.LF ..V......M ..F..H..TT I...R..AS. ...ETY.RRR V.Q.GH....
Wyoming_AF033820    .......V.F ..I.LL.... .....A.WKV T......GS. ...KKF.H.. .SR..T...A

BRA1                QYRIHLADAT GGSEDKYNKP KCSKRSYSGG LKGFNRQPKN WKNSTERSEV NSTGLGEHME
BRA2                ..SV...GV. ..L......L .Y...N.N.. S.E..KR..S .ETLIDA.GK ....P.PS.G
DLV9-7_HM141922     ..HAY...E. H..G..S.MR .L.RNNWN.E SEEY..RQ.. ..RLIK..GE .YN-------
DV10-3_HM141910     ..HAY...E. H..G..S.MR .LPRNNWN.E PEEY..RQ.. ..RLIK..GE .YNTHEDN.G
IRE_F2_JX480631     ...V...SVN D...G.F.RQ RAF.N.W..E SEEYSSRR.H YEK..KEFGK SFN..ADYE.
IRE_F3_JX480632     ...V...SVN D...G.FSRQ RAF.N.W..E SEEYSSRR.H YEK..KEFGK SFN..ADYE.
IRE_F4_JX480633     ...V...SVN D...G.F.RQ RAF.N.W..E SEEYSSRR.H YEK..KEFGK SFN..ANYE.
IRE_H3_JX480634     ...V...SVN D...G.FSRQ RAF.N.W..E SEEYSSRR.H YEK..KEFGK SFN..ANYE.
ITA_DE_KM247554     ...V...GVN D...G.FSRQ RAF.N.W..E SEEYSSRR.H YEA..KKFGE SFN..ADTK.
ITA_SA_KM247555     ...V...SVN D...G.F.RQ RAF.N.W..E SEEYSSRR.H YET..KEFGE SFN..ANNK.
Liaoning_AF327877   ..HAY...E. H..G..S.MR .L.RNNWN.E SEEY..RQ.. ..KLIK..GE .YNTHEDN.G
Miyazaki_JX003263   ..N....NV. V...EEF.TQ NNFRNNWN.D S.VYDKLQ.H .RRL.K..GE DWHIP.VNEG
Wyoming_AF033820    .HN....GV. ...G...Y.Q .Y.RNDWN.E SEEY..R..S .VK.I.AFGE SYISEKTKG.

BRA1                TSHLSIAIAE -NS-VSGDNA QRTSTSLKIQ SEGGNIYDCC IKAQEGTLAI PCCGFPLWLF
BRA2                ITLP.M..G. TK.-.A.... .QA.AN.E.R .......... .......... ..........
DLV9-7_HM141922     -MGRLVTT.A EKK-NV.V.P HQG.LN.E.. .......... .......... .........L
DV10-3_HM141910     .MGHL.TT.A EKK-NV.E.P HQG.LN.E.. .......... ....G..... .........L
IRE_F2_JX480631     ITPAF..PNV KKD-AGEK.P SQG.L..E.. .......... .......... ..........
IRE_F3_JX480632     ITPAF..PNV KKD-AGEK.P SQG.L..E.. .......... .......... ..........
IRE_F4_JX480633     ITPAF..PNV KKD-AGEK.P SQG.L..E.. .......... .......... ..........
IRE_H3_JX480634     ITPAF..PNV KKD-AGEK.P SQG.L..E.. .......... .......... ..........
ITA_DE_KM247554     ITPTF..PNV KKD-AGEK.P SQG.L..E.. .......... .......... ..........
ITA_SA_KM247555     ITPTF.PPNV KKD-AGEK.P SQG.L..E.. .......... .......... ..........
Liaoning_AF327877   .MGRLVTT.A EKK-NV.V.P HQG.LN.E.. .......... .......... .........L
Miyazaki_JX003263   ...HGTTTGA KKK-HI.G.P .QG.LD.E.. .T........ ........T. ..........
Wyoming_AF033820    I.QPGA..N. HKNGSG.N.P HQG.LD.E.R .......... .......... ..........

BRA1                WGLIIIMGRL FGYGLRGIAM ILSYCEKGLT MLFIMLKRLC DFIGRALNPA TSHVSMPQYV
BRA2                ..FV..I... L......... ..L..G.... ....?..... .S........ ..........
DLV9-7_HM141922     .....L.... L...F....K FIMILG...N VIITG.RK.. .Y..KM.... ....T....D
DV10-3_HM141910     .....L.... L........K .IMILG...N VIITG.RK.. .Y..KM.... ....T....D
IRE_F2_JX480631     .....L.... I......L.K .IMFSGRVIL LSINW.RKGL .YV.KM...S K...T..C.D
IRE_F3_JX480632     .....L.... I......L.K .IMFSGRVIL LSINW.RKGL .YV.KM...S K...T..C.D
IRE_F4_JX480633     .....L.... I......L.K .IMFSGRVIL LSINW.RKGL .YV.KM...S K...T..C.D
IRE_H3_JX480634     .....L.... I...F..L.K .IMFSGRVIL LSINW.RKGL .YV.KM...S K...T..C.D
ITA_DE_KM247554     .....L.... I......L.K .IMFSGRVIL LSINW.RKGL .YV.KM...S K...T..C.D
ITA_SA_KM247555     .....L.... I......L.K .IMFSGRVIL LSINW.RKGL .YV.KM...S K...T..C.D
```

```
Liaoning_AF327877    ......L... L........K .IMILG...N VIITG.RK.. .Y..KM.... ....T....D
Miyazaki_JX003263    ...L...... L....K.L.I M.QIIG...Y G.LN.I.QIF .YM.N.FS.P K..I.....I
Wyoming_AF033820     ...V..V..I A......L.V .IRI.IR..N LI.EIIRKML .Y.......G ..........


BRA1                 --
BRA2                 --
DLV9-7_HM141922      V-
DV10-3_HM141910      V-
IRE_F2_JX480631      I-
IRE_F3_JX480632      I-
IRE_F4_JX480633      I-
IRE_H3_JX480634      I-
ITA_DE_KM247554      I-
ITA_SA_KM247555      I-
Liaoning_AF327877    V?
Miyazaki_JX003263    --
Wyoming_AF033820     --
```

Supplementary Figure S6: Phylogenetic trees of Brazilian field sequences and foreign sequences. Each tree was performed based a genome region or gene: A. LTR; B. Gag; C. Pol; D. Env; E. Env protein. Used sequences: Liaoning (AF327877), Wyoming (AF033820), Miyazaki 2011-A (JX003263), IRE F2 (JX480631) IRE F3 (JX480632), IRE F4 (JX480633), IRE H3 (JX480634), ITA SA (KM247555), ITA DE (KM247554), and LTR sequences from China (KY411969-76; KY412097-98).

A.

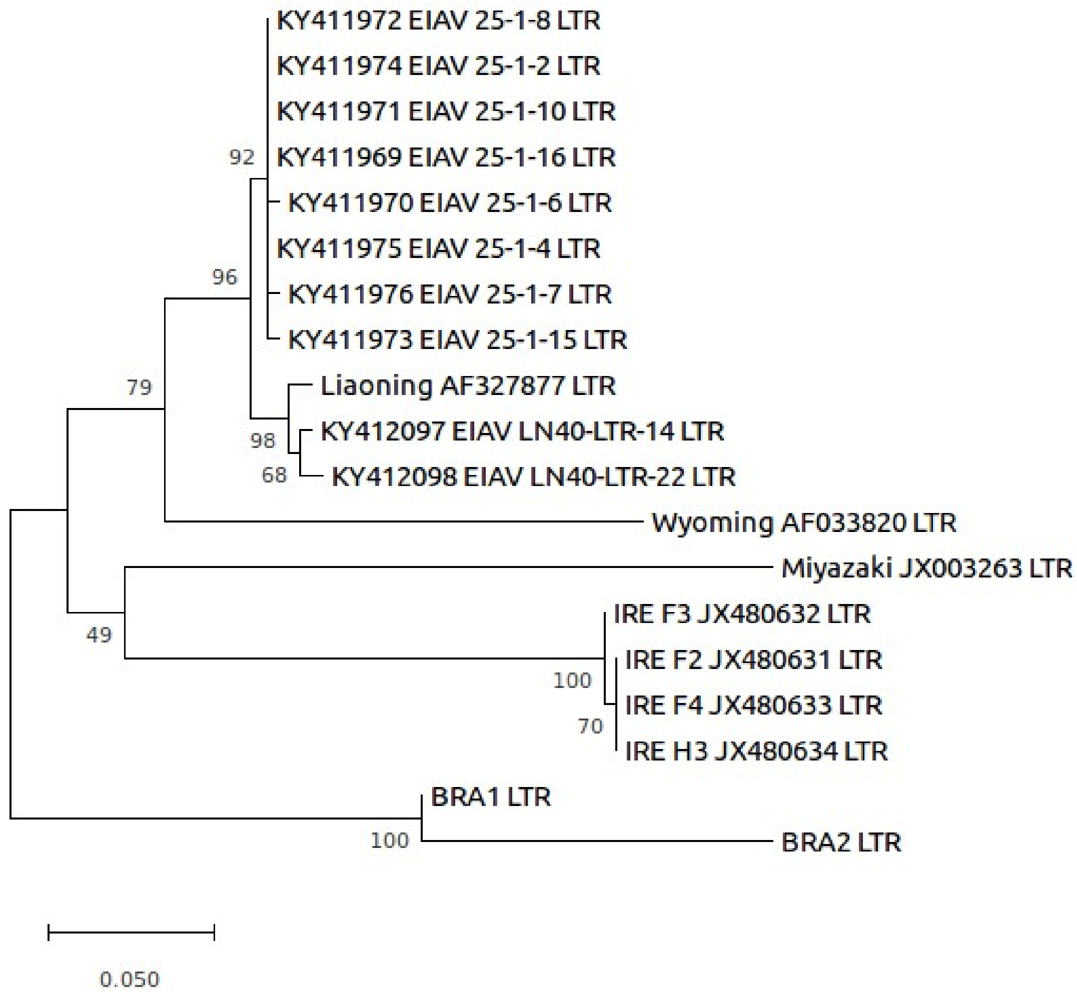

B.

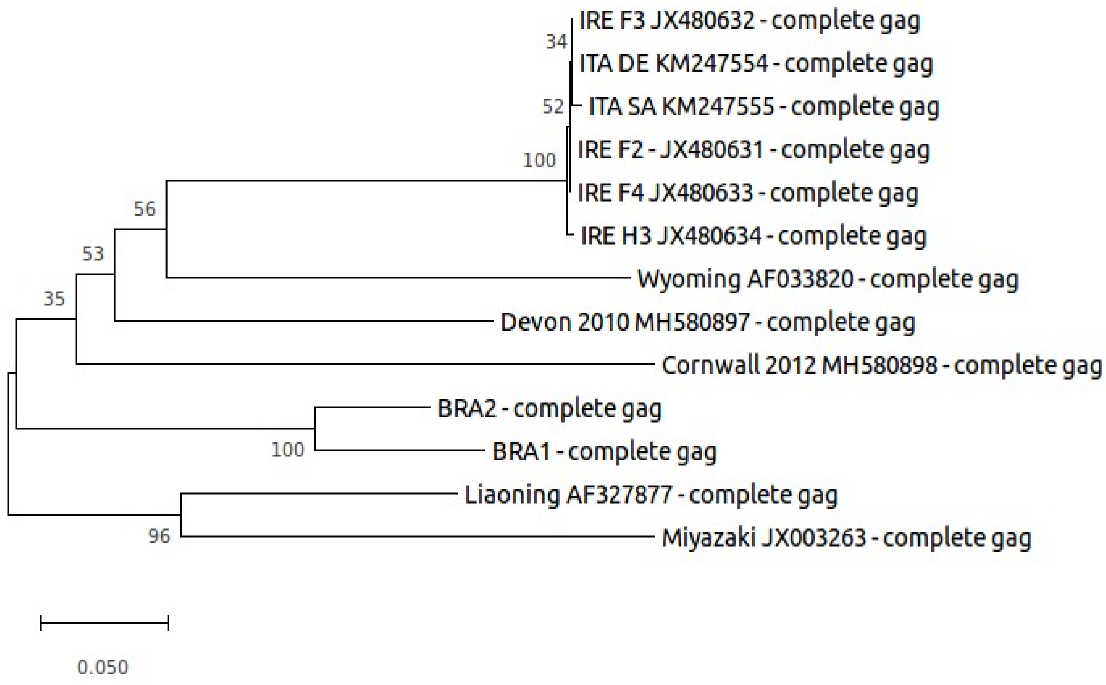
IRE F3 JX480632 - complete gag
ITA DE KM247554 - complete gag
ITA SA KM247555 - complete gag
IRE F2 - JX480631 - complete gag
IRE F4 JX480633 - complete gag
IRE H3 JX480634 - complete gag
Wyoming AF033820 - complete gag
Devon 2010 MH580897 - complete gag
Cornwall 2012 MH580898 - complete gag
BRA2 - complete gag
BRA1 - complete gag
Liaoning AF327877 - complete gag
Miyazaki JX003263 - complete gag
34
52
100
56
53
35
100
96
0.050

C.

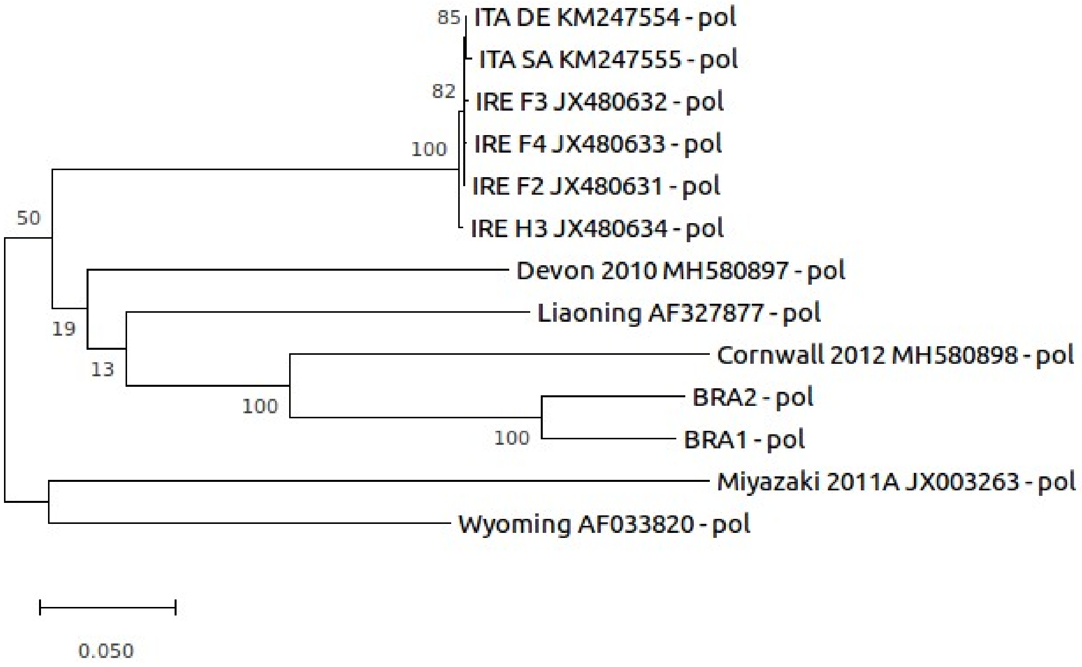
85
ITA DE KM247554 - pol
ITA SA KM247555 - pol
82
IRE F3 JX480632 - pol
IRE F4 JX480633 - pol
100
IRE F2 JX480631 - pol
IRE H3 JX480634 - pol
50
Devon 2010 MH580897 - pol
Liaoning AF327877 - pol
19
13
Cornwall 2012 MH580898 - pol
100
BRA2 - pol
100
BRA1 - pol
Miyazaki 2011A JX003263 - pol
Wyoming AF033820 - pol
0.050

D.

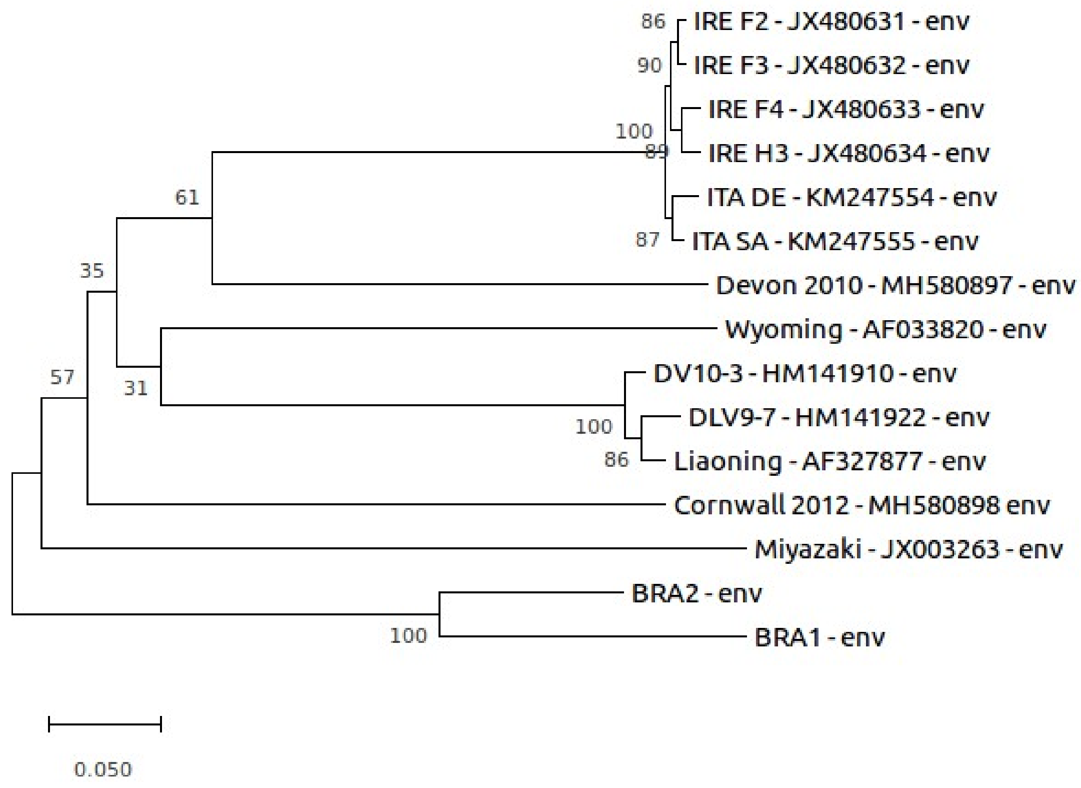

IRE F2 - JX480631 - env
IRE F3 - JX480632 - env
IRE F4 - JX480633 - env
IRE H3 - JX480634 - env
ITA DE - KM247554 - env
ITA SA - KM247555 - env
Devon 2010 - MH580897 - env
Wyoming - AF033820 - env
DV10-3 - HM141910 - env
DLV9-7 - HM141922 - env
Liaoning - AF327877 - env
Cornwall 2012 - MH580898 env
Miyazaki - JX003263 - env
BRA2 - env
BRA1 - env
86
90
100
89
87
61
35
57
31
100
86
100
0.050

E.

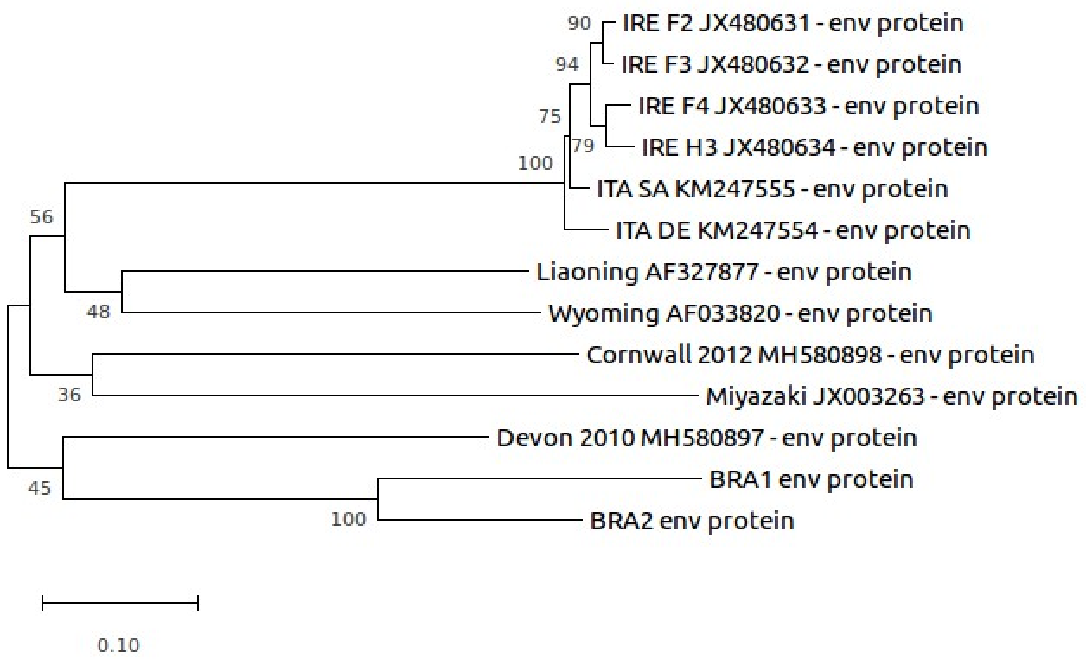
IRE F2 JX480631 - env protein
IRE F3 JX480632 - env protein
IRE F4 JX480633 - env protein
IRE H3 JX480634 - env protein
ITA SA KM247555 - env protein
ITA DE KM247554 - env protein
Liaoning AF327877 - env protein
Wyoming AF033820 - env protein
Cornwall 2012 MH580898 - env protein
Miyazaki JX003263 - env protein
Devon 2010 MH580897 - env protein
BRA1 env protein
BRA2 env protein
90
94
75
79
100
56
48
36
45
100
0.10

Supplementary Figure S7: Multiple alignment of partial *gag* sequences from Bahia (Brazil) and BRA1 and 2 from Pantanal (Brazil).

```
KC213783.1    CCTAGAGGAT ATACTACTTG GGTGAATCCC ATACAGACAA ATGGTCTATT AAATGAAGCT
KC213784.1    .......... .......... .......... .......... .......... ..........
KC213785.1    .......... .......... .......... .......... .......... ..........
KC213786.1    .......... .......... .......... .......... .......... ..........
KC213787.1    .......... .......... .......... .......... .......... ..........
KC213788.1    .......... .......... .......... .......... .......... ..........
KC213789.1    .......... .......... .......... .......... .......... ..........
KC213790.1    .......... .......... .......A.. .......... .......... .......C..
BRA1 - Gag    ..C....... .C..C..C.. ...A...A.T ......CA.. ..AA.T.G.. ......G..A
BRA2 - Gag    ..C....... .C..C..C.. ...A...A.T ......CA.. .CAA...G.. ......G..A

KC213783.1    AGTCAAAACT TATTTGGGAT ATTATCAGTA GACTGTACTT CTGAAGAAAT GAATGCATTT
KC213784.1    .......... .......... .......... .......... .......... ..........
KC213785.1    .......... .......... .......... .......... .......... ..........
KC213786.1    .......... .......... .......... .......... .......... ..........
KC213787.1    .......... .......... .......... .......... .......... ..........
KC213788.1    .......... .......... .......... .......... .......... ..........
KC213789.1    .......... .......... .......... .......... .......... ..........
KC213790.1    .......... .......... .......... .......... .......... ......C...
BRA1 - Gag    ...GT..... .G.....A.. T..G...... ..T..C..A. .G........ ..........
BRA2 - Gag    ...GT..... .G.....A.. ...G...... ..T..C..A. ....G..... ..........

KC213783.1    TTGGATGTGG TACCTGGCCA GGCAGGACAA AAGCAGATAT TACTTGATGC AATTGATAAA
KC213784.1    .......... .......... .......... .......... .......... ..........
KC213785.1    .......... .......... .......... .......... .......... ..........
KC213786.1    .......... .......... .......... .......... .......... ..........
KC213787.1    .......... .......... .......... .......... .......... ..........
KC213788.1    .......... .......... .......... .......... .......... ..........
KC213789.1    .......... .......... .......... .......... .......... ..........
KC213790.1    .......... .......... .......... .......... .......... ..........
BRA1 - Gag    C.A....... ....A..A.. A......... ..A..AG.T. .G......TT .C.A..C..G
BRA2 - Gag    C.A....... .G..A..A.. A........G ..A..AG.TC ........TT GC.G.....G

KC213783.1    ATAGCAGATG ATTGGGATAA TAGACATCCA TTACCGAATG CTCCACTGGT GGCACCACCA
KC213784.1    .......... .......... .......... .......... .......... ..........
KC213785.1    .......... .......... .......... .......... .......... ..........
KC213786.1    .......... .......... .......... .......... .......... ..........
KC213787.1    .......... .......... .......... .......... .......... ..........
KC213788.1    .......... .......... .......... .......... .......... ..........
KC213789.1    .......... .......... .......... .......... .......... ..........
KC213790.1    .......... .......... .......... .......... .......... ..........
BRA1 - Gag    ..G..T..A. .......C.. ...G...... .....A..CC .A..CT.... ...G..GG..
BRA2 - Gag    .....G..A. .......C.. .......... ..G..A..CC .A..TT.... A..G..GG..

KC213783.1    CAAGGGCCTA TTCCCATGAC AGCAAGGTTT ATTAGAGGTT TAGGAGTACC TAGAGAAAGA
KC213784.1    .......... .......... .......... .......... .......... ..........
KC213785.1    .......... .......... .......... .......... .......... ..........
KC213786.1    .......... .......... .......... .......... .......... ..........
KC213787.1    .......... .......... .......... .......... .......... ..........
KC213788.1    .......... .......... .......... .......... .......... ..........
KC213789.1    .......... .......... .......... .......... .......... ..........
KC213790.1    .......... .......... .......... .......... .......... ..........
BRA1 - Gag    .....A.... ....T..... G.....A... ........G. .......T.. .........G
BRA2 - Gag    .....A.... ....T..... G..G..A... ........A. .......T.. .........G
```

```
KC213783.1    CAGATGGAGC CTGCTTTTGA TCAGTTTAGG CAGACATATA GACAATGGAT AATAGAAGCC
KC213784.1    .......... .......... .......... .......... .......... ..........
KC213785.1    .......... .......... .......... .......... .......... ..........
KC213786.1    .......... .......... .......... .......... .......... ..........
KC213787.1    .......... .......... .......... .......... .......... ..........
KC213788.1    .......... .......... .......... .......... .......... ..........
KC213789.1    .......... .......... .......... .......... .......... ..........
KC213790.1    .......... .......... .......... .......... .......... ..........
BRA1 - Gag    ........A. .A..C..... ......C..A .....T.... ....G..... ...T..G..G
BRA2 - Gag    ..A.....A. .A..C..... ......C..A ..A..T.... .......... ...T..G...

KC213783.1    ATGTCAGAAG GCATCAAAGT GATGTCGGTA AAA
KC213784.1    .......... .......... .......... ...
KC213785.1    .......... .......... .......... ...
KC213786.1    .......... .......... .......... ...
KC213787.1    .......... .......... .......... ...
KC213788.1    .......... .......... .......... ...
KC213789.1    .......... .......... .......... ...
KC213790.1    .......... .......... ....ATT.G. ...
BRA1 - Gag    ...A...... .A..A...A. A...ATT.G. ..G
BRA2 - Gag    ...A....G. .A..A...A. A...ATA.G. ..G
```

Supplementary Figure S8: Phylogenetic tree of partial *gag* sequences from Bahia (Brazil), BRA1 and 2 from Pantanal (Brazil), and field sequences of other countries.

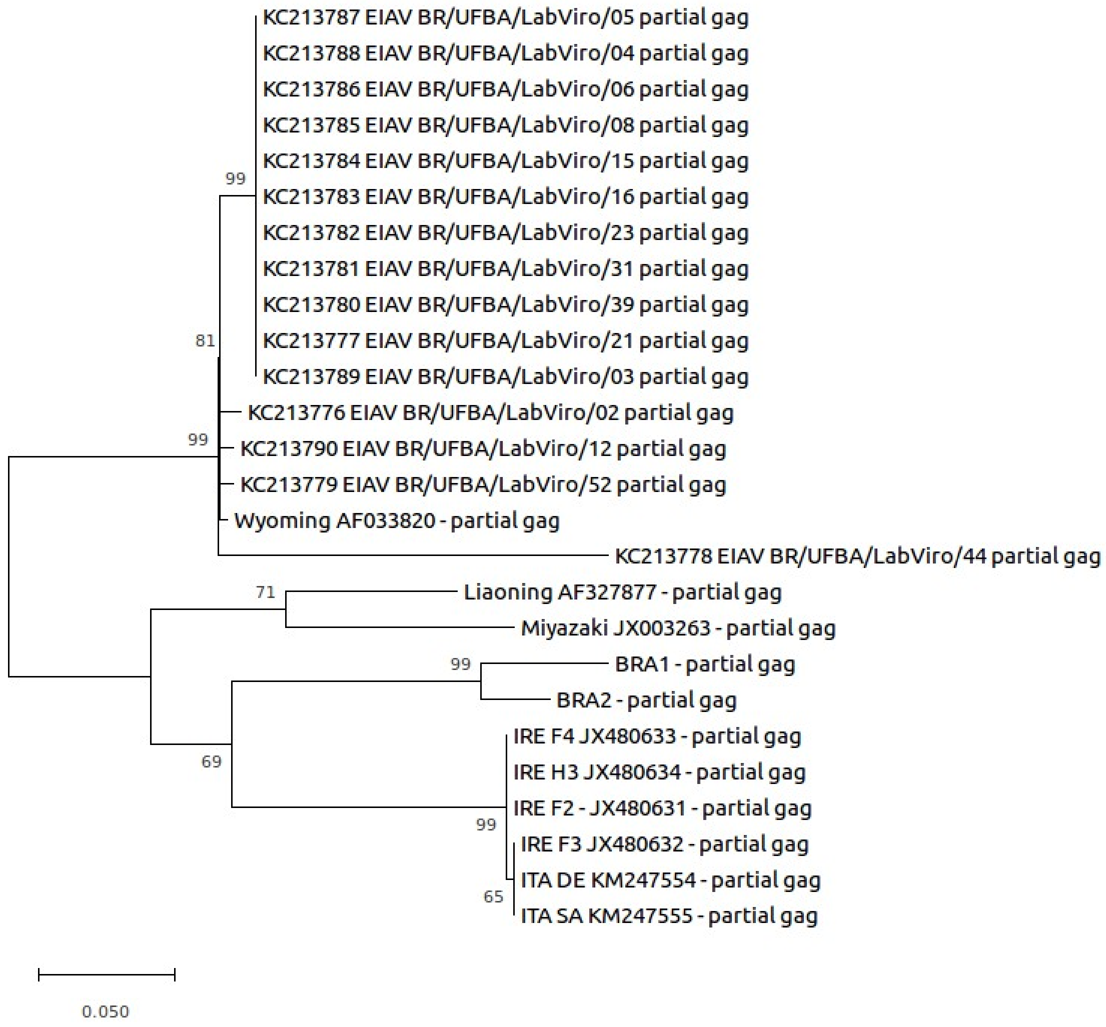

Supplement: Supplementary file 1 [file viruses-12-00207-s001.pdf]
